# Photosensitizing Au(I) Catalysis With Coumarins

**DOI:** 10.1002/chem.71185

**Published:** 2026-06-25

**Authors:** Liliana Capulín Flores, Jacob J. van der Wal, Johannes E. M. N. Klein

**Affiliations:** ^1^ Stratingh Institute for Chemistry University of Groningen Groningen The Netherlands

**Keywords:** coumarin, gold complex, photocatalysis, photosensitizer, triplet excited state

## Abstract

Gold catalysis shows versatility in a wide range of challenging transformations, wherein light‐driven reactions open new pathways. Understanding the factors that impact photosensitization is essential for harnessing the reactivity of excited states in gold photoredox and energy transfer catalysis. We use a Au(I) complex attached to a 3‐amino coumarin chromophore (**1**) to investigate the impact of intramolecular photosensitization on its photophysical and photochemical properties. The potential of **1** to mediate light‐driven electron‐ or energy‐transfer processes was tested in two representative cross‐coupling reactions. **1** performs moderately in these reactions, which we attribute to the emergence of competing pathways, as indicated by photophysical studies. At 298 K **1** emits weakly, which is attributed to the coumarin chromophore based on UV‐vis absorption and emission spectroscopy, and supported by DFT calculations. Although advantageous in coupling the coumarin to the gold complex, self‐quenching by the amide group results in a reduction of the emission from **1** relative to that of 3‐amino coumarin. At 77 K **1** shows phosphorescence, which is absent in the coumarin alone, indicating intramolecular energy transfer is possible. These data indicate that photochemical activation of gold catalysis holds potential, but effective energy transfer between chromophore and catalyst remains a challenge.

## Graphical abstract

A Au(I) complex with a tethered coumarin fragment was synthesized and characterized by UV‐vis and emission spectroscopy. Its ability to facilitate photo‐driven cross‐coupling reactions through single‐electron and energy transfer events was evaluated.

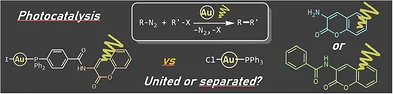

## 1 Introduction

Gold catalysis has emerged as a promising field in both heterogeneous [1, 2, 3, 4, 5, 6] and homogeneous [7, 8, 9, 10, 11, 12, 13, 14] processes in which the unique properties of gold have been leveraged to drive organic transformations. In molecular catalysis, both Au(I) and Au(III) complexes have been exploited in organic synthesis, in particular for the activation of unsaturated bonds [7, 8, 9, 10, 11, 12, 13, 14]. In contrast, Au(I) or Au(III) complexes are scarcely considered in redox catalysis, likely due to the difficulty in switching redox states thermally to, for example, drive reactions with simple terminal oxidants [9, 15, 16, 17, 18, 19, 20]. More recently, photochemical activation has opened up new possibilities for driving redox events at metal centers, typically through the use of an added photocatalyst. Gold photoredox catalysis has therefore established its relevance as a synthetic strategy for mediating processes under mild conditions [21, 22, 23, 24, 25]. Less explored, energy transfer (EnT) has paved its way into gold photocatalysis as a non‐redox mechanism that uses the energy of an excited state to promote substrate conversion [26, 27, 28, 29, 30, 31]. While mechanistically distinct, both strategies have enabled access to complex transformations that would otherwise be unachievable under thermal conditions [27, 30]. In some cases, such conversions occur without the need for an external photocatalyst [32], where an intermediate generated from the gold catalyst [33, 34] or the gold catalyst itself [28, 35, 36, 37] behaves as a photocatalyst.

Phosphine‐based gold complexes, usually combined with metal‐based or organic photocatalysts, have been extensively employed in light‐driven catalysis [38, 39, 40, 41, 42]. Notably, the archetypal Ph_3_PAuCl pre‐catalyst exhibits light‐driven reactivity at low temperatures (77 K) in the UV region [43]. While this indicates potential to behave as a photocatalyst *per se*, the incorporation of chromophore units near the metal center has been explored in a few cases to harness low‐energy photoactivation pathways (*vide infra*), a strategy that has been more widely employed in transition metal catalysis [44, 45, 46, 47, 48].

In 2019, some of us reported that a triphenylphosphine vinyl Au(I) species, accessible *in situ* by reacting Ph_3_PAuCl with an *o*‐alkynyl phenol, afforded the functionalization of benzofurans under irradiation in the presence of a diazonium salt without the need for an additional photocatalyst [34]. Related reactions reported by Fensterbank and co‐workers [33, 49, 50], also highlighted the potential involvement of triplet energy transfer (EnT) as a key mode of activation in these reactions, prompting us to further explore the reactivity of triplet excited states in Au(I) photochemistry. We wondered if such photoreactivity could be boosted by incorporating a photoactive moiety, with readily accessible triplet states, into the structure of the benchmark Ph_3_PAuCl catalyst.

Au(I) complexes featuring covalently attached photoactive fragments have found applications in the realms of photodynamic therapy [51, 52, 53, 54, 55] (e.g., trackable therapeutic agents, singlet oxygen generation) and catalysis, wherein one of the coordinated ligands incorporates a photosensitizer moiety [56, 57, 58, 59, 60]. For photo‐driven cross‐coupling catalysis, phosphine Au(I) complexes with tethered metal‐based photosensitizers have been investigated (e.g. [Ru(bpy)_3_]^2+^), aiming to improve photosensitization by taking advantage of the redox properties of [Ru(bpy)_3_]^2+*^ to drive single electron transfer (SET) processes [56]. However, modest improvement was observed for the arylative Meyer–Schuster rearrangement of propargyl alcohols when compared to the separate metal and photosensitizer units [56]. This observation was rationalized as a consequence of deactivation pathways stemming from metal‐metal through‐space interactions [56, 57]. Attempts to reduce the extent of through‐space interaction by changing the orientation of the photosensitizer unit resulted in a slight improvement in catalytic performance (Chart 1a, b) [57].

**CHART 1:**
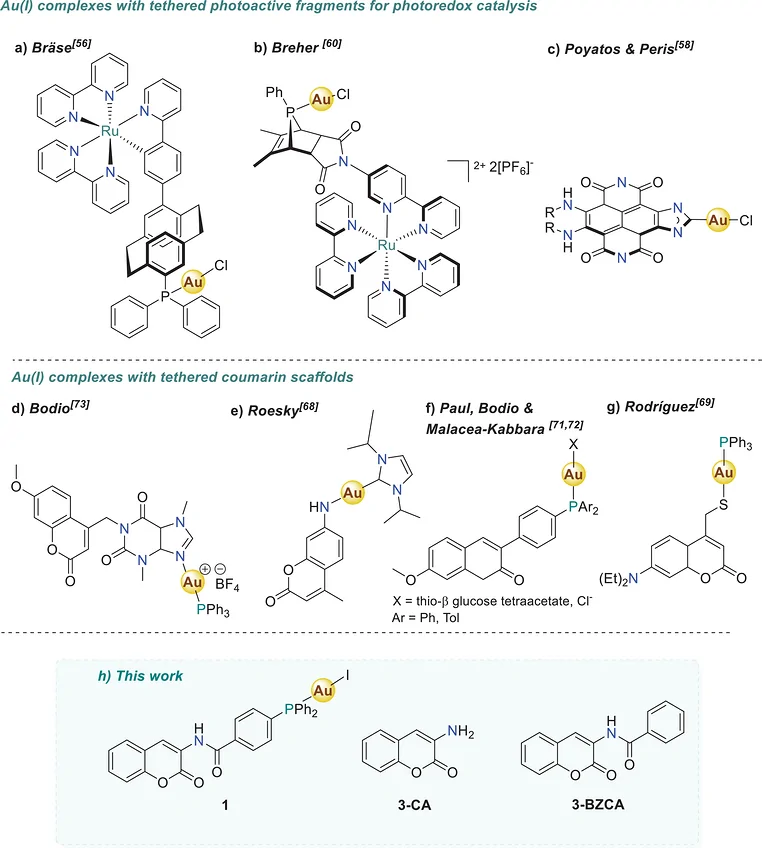
Representative examples of Au(I) complexes featuring photoactive fragments (a‐g) and complex **1** studied in this work (h).

Compared to tethered metal‐based fragments, organic chromophores covalently attached to Au(I) species have displayed more encouraging results. Peris and co‐workers [58] reported the photocatalytic activity of an *N*‐heterocyclic carbene Au(I) complex furnished with a naphthalene‐di‐imide (NDI) moiety (Chart 1c). This complex acts as a dual metallophotoredox catalyst for the coupling reaction of aryldiazonium salts with different nucleophiles (trimethylsilylethynylbenzene, mesitylene), a process that proceeds via SET [58]. The authors found that the Au(I)‐NDI catalyst (10 mol%) outperforms the activity of the archetypical Ph_3_PAuCl (10 mol%)/[Ru(bpy)_3_]^2+^ (0.5 mol%) dyad [58]. Furthermore, Au‐NDI proved to be an efficient photosensitizer for the generation of singlet oxygen (^1^O_2_) by triplet‐triplet EnT, which was ultimately harnessed in the endo‐peroxidation of organic compounds [58]. Although no data were reported regarding the activity of both Au(I) catalyst and NDI photocatalyst as separate entities, this example manifests the potential of merging an organic chromophore and a gold‐based catalyst in the same unit.

Here, aiming to exploit the reactivity of excited states in gold photocatalysis, we prepare and study the photophysical properties of a new phosphine‐based coumarin Au(I) complex and evaluate its reactivity in light‐driven cross‐coupling reactions. We selected the coumarin scaffold as its inclusion into the structure of Ir(III) complexes as a light‐harvesting unit has been shown to promote long‐lived triplet excited states [61, 62]. Moreover, various examples in the literature demonstrate the potential of coumarins to act as triplet photosensitizers, thereby opening opportunities to explore their reactivity in EnT catalysis [63, 64, 65, 66, 67]. We envision that a coumarin covalently bound to a Ph_3_PAuCl‐derived species would promote the formation of long‐lived (triplet) excited states that could be leveraged in gold photocatalysis. Au(I) coumarin complexes have, indeed, been previously investigated for their particular photophysical properties (Chart 1e,g) [68, 69] as well as their ability to behave as smart probes in medicinal chemistry (Chart 1d,f) [70, 71, 72, 73, 74]. However, little is known about their potential to promote catalytic properties for light‐driven reactions, an aspect we investigate here (Chart 1, bottom). Our findings indicate that tethering the coumarin to the benchmark Ph_3_PAuCl catalyst provides little improvement in catalytic performance, demonstrating the challenges associated with covalently attaching chromophores to the structure of a metal‐based catalyst. Notably, limited success has also been found for related approaches shown in Chart 1 where catalytic activity improves, nevertheless only moderately.

## 2 Results & Discussion

### 2.1 Synthesis and Characterization

Complex **1** was synthesized following the procedure depicted in Scheme 1. *Via* a two‐step one‐pot reaction, benzoic acid triphenylphosphine gold(I) chloride^1^ was transformed into the corresponding activated pyridinium ester upon reacting with 2‐chloro‐1‐methylpyridium iodide ‘Mukaiyama's reagent’ [75] (1.5 eq.) and triethylamine (5 eq.). The addition of 1 equivalent of 3‐aminocoumarin (**3‐CA**) and subsequent stirring at 30 °C for 24 h resulted in the formation of the expected product in moderate yields (65%–73%) [76]. The chloride ligand in the gold precursor proved to be labile, as analysis of the composition of **1** by X‐ray and high‐resolution mass spectrometry (HRMS, Figure S7) indicated the formation of the corresponding iodide derivative *via* ion exchange with the iodide provided by the Mukaiyama reagent.

**SCHEME 1:**
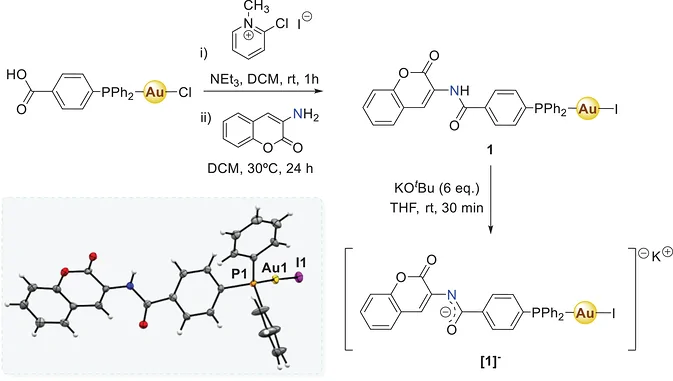
Synthesis of complex **1** and **[1]** ^−^. Thermal ellipsoid of **1** shown at the 50% probability level.

The ^1^H NMR spectrum of **1** exhibits two singlets at 8.83 and 8.85 ppm assigned to the amide proton and the methine group of the coumarin moiety, respectively, confirming the successful incorporation of the chromophore into the structure of the gold(I) precursor (Figure S1). Similarly, in the ^13^C NMR spectrum, a signal in the most downfield region at 165 ppm is consistent with the presence of the amide group in **1**, while the resonance of the methine group is observed at 124.1 ppm (Figure S2). In the ^31^P{^1^H} NMR spectrum, the singlet at 38.6 ppm is assigned to the phosphorus in the neutral ligand. The FT‐IR spectrum displays two distinct C═O stretching bands in the solid state, at 1663 and 1705 cm^−1^, corresponding to the carbonyl groups of the amide and lactone functionalities, respectively (Figure S6).

Suitable crystals for single‐crystal X‐ray diffraction were obtained from the slow diffusion of pentane into a CH_2_Cl_2_ solution of **1** [77]. In the molecular structure of **1**, the geometry around the metal center is nearly linear (176.4°), with the neutral and anionic ligands positioned *trans* to each other. The bond distance Au(1)‐P(1) in **1** (2.252(1) Å) falls within the range reported for other representative mononuclear Au(I) phosphine species: 2.235(3) Å in Cl‐Au‐PPh_3_ [78], 2.2385(7) Å in Cl‐Au‐P(tol)_2_(Ph‐8‐methoxycoumarin) [71]. In the case of the Au(1)‐I(1) bond length (2.5658 (5) Å), its magnitude is comparable to that found in I‐Au‐P(Cy)_3_ (2.577 Å) [79].

### 2.2 Photophysical Characterization

Photophysical measurements were recorded in degassed acetonitrile solutions unless stated otherwise, and data are summarized in Table 1. The UV‐vis absorption spectrum of **1** at room temperature exhibits a broad absorption at 328 nm with a molar absorptivity of 29 500 M^−1^ cm^−1^, which arises from π‐π* electronic transitions centered on the coumarin fragment (Figure 1a), consistent with TDDFT calculations (*vide infra*). As expected, a similar feature is observed in the free chromophore **3‐CA** (λ_max_ = 323 nm, ε = 54 000 M^−^^1^ cm^−^^1^).

**FIGURE 1:**
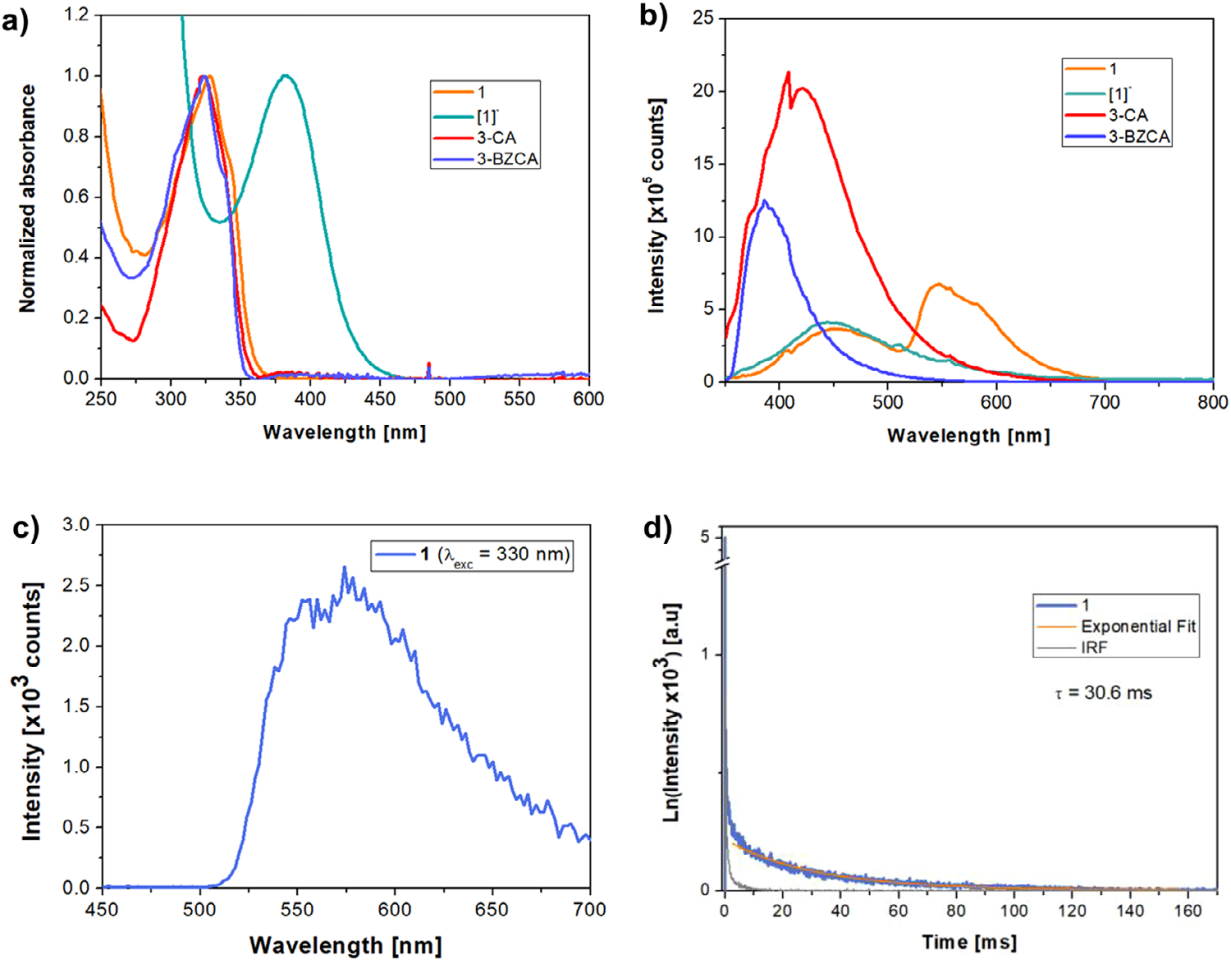
(a) UV‐vis absorption spectra of **1** [7.6 × 10^−4^ M], **[1]** ^–^ [7.6 × 10^−4^ M], **3‐BZCA** and **3‐CA** [2 × 10^−6^ M] in MeCN (**1**, **3‐BZCA** and **3‐CA**) and THF (**[1]** ^−^) at room temperature. (b) Emission spectra in frozen matrix of **1** (λ_exc_ = 330 nm), **[1]** ^−^ (λ_exc_ = 340 nm), **3‐CA** (λ_exc_ = 330 nm) and **3‐BZCA** (λ_exc_ = 335 nm) at 77 K in MeCN. (c) Gated phosphorescence emission of **1** (λ_exc_ = 330 nm) in frozen MeCN solution. (d) Emission decay (λ_exc_ = 330 nm) at 77 K in MeCN, instrument response function (IRF) in gray.

**TABLE 1:** Photoluminescence properties of **1**, **[1]^−^**, **3‐BZCA** and **3‐CA**.

Compound	λ_max_ (nm)	ε = M^−1 ^cm^−1^	λ_em_ (λ_exc_) (nm)^a^	λ_em_ (λ_exc_) (nm)^b^	*E*_0‐0_ (eV)^c^	*E*_ox_ (PC^+•^/PC) [V] versus Fc^+/0^	E*_ox_ (PC^+•^/PC*) [V] versus Fc^+/0^
**1**	328, 331^d^	29 500, 31 500^d^	375 (330), 368, 560 (330)^d^	450, 550 (330)	3.54, 3.52^d^	1.03^d^	−2.54, −2.49^d^
**[1]** ^−^ ^e^	383	57 100	426 (340)	448 (340)	3.08	0.28	−2.80
**3‐BZCA**	323	45 800	370 (329)	420 (335)	3.60	0.95	−2.65
**3‐CA**	323	54 000	380 (330)	387 (330)	3.54	0.84	−2.70

^a^ In solution at 298 K.

^b^ In frozen matrix at 77 K.

^c^ The adiabatic excitation energy *E*_0,0_ was determined from the intersection between the absorption and emission spectra at 298 K.

^d^ Measured in dichloromethane.

^e^ Measured in tetrahydrofuran.

**FIGURE 2:**
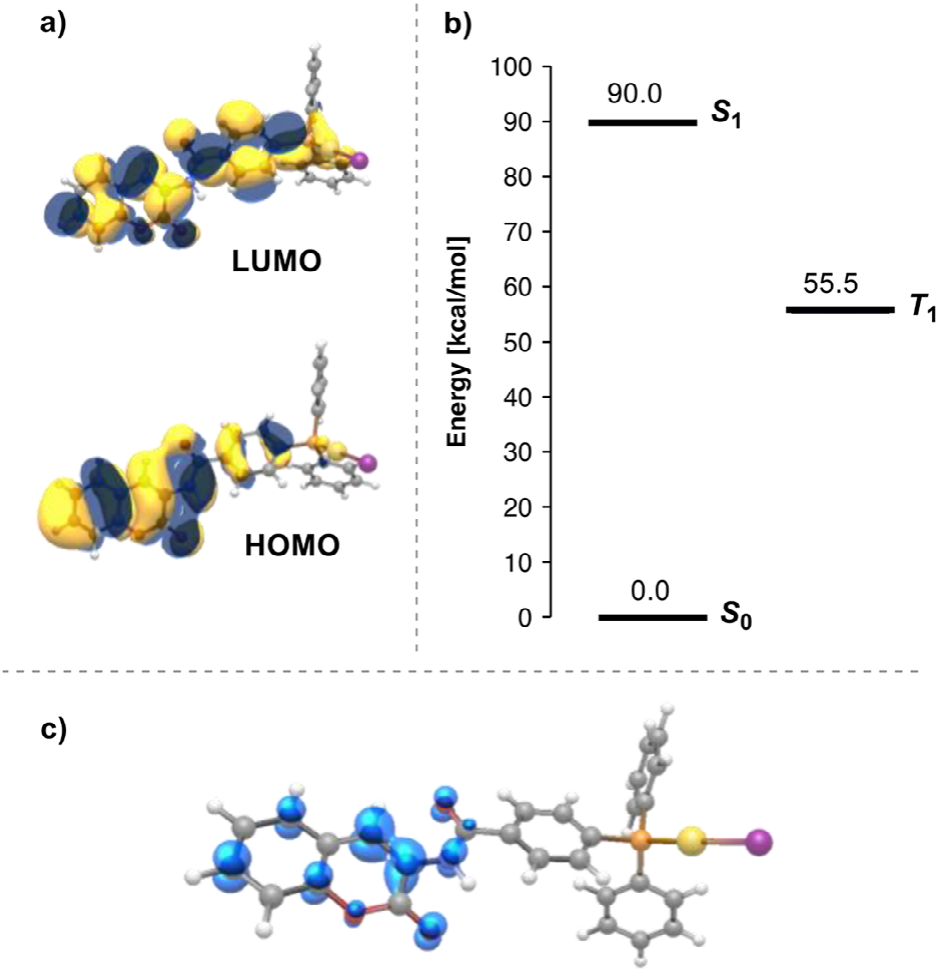
(a) Frontier orbitals of **1** (isovalue = 0.03). (b) Energy profile of the excited state *S_1_* and *T_1_*. (c) Spin density plot for *T_1_* (isovalue =0.01). All data was computed at CAM‐B3LYP‐D3(BJ)‐def2‐TZVP/CPCM(MeCN)//TPSS‐D3(BJ)/def2‐TZVP/CPCM(MeCN) level of theory.

Although covalent attachment of the coumarin moiety to gold has minimal influence on the absorption spectrum of the chromophore, it has a pronounced effect on its emission. Complex **1** is weakly emissive at room temperature, with an emission band at 375 nm (λ_exc_ = 330 nm), whereas the chromophore unit itself shows a stronger emission at 380 nm (λ_exc_ = 330 nm, Figure S10). The emission spectrum of **1** in CH_2_Cl_2_ (λ_exc_ = 330 nm) exhibits, in addition to the shorter wavelength emission at 369 nm, a low‐intensity band at 570 nm (Figure S11).

The 3‐benzoylamide coumarin (**3‐BZCA**) scaffold, a fragment that more closely mimics the chemical environment of the attached chromophore, was synthesized to evaluate the effect of the amide functionality on the photophysics of the coumarin in **1**. This compound was prepared by condensing **3‐CA** with benzoyl chloride. The UV‐vis absorption spectrum of **3‐BZCA** is essentially the same as that of **1** and **3‐CA,** with an absorption band at λ_max_ = 323 nm (ε = 45 800 M^−1^ cm^−^^1^) (Figure 1a). Its weak fluorescence at room temperature (λ_em_ = 370, λ_exc_ = 329 nm) compared to the amino‐chromophore **3‐CA** suggests that the amide group may be involved in fluorescence quenching *via* photoinduced electron transfer, as has been observed in aromatic amino acids when converted into their corresponding amide derivatives [80]. Analog spectroscopic signatures are reported for related phosphine Au(I) coumarin complexes, wherein fluorescence is observed at room temperature (R‐Au‐PPh_3_, R = 4‐(thiolmethyl)‐7‐(diethylamino)‐2H‐chromen‐2‐one: λ_abs_ = 378 nm, λ_em_ = 450 nm [69]; R‐Ph‐Ptol_2_‐Au‐Cl, R = 7‐methoxy‐2H‐chromen‐2‐one: λ_abs_ = 350 nm, λ_em_ = 432 nm [72]) characterized by short‐lived excited states ranging from 2–4 ns [69, 81], similar to what has been reported for the free coumarin chromophores [82, 83]. Given the weak emission from the compounds herein investigated at room temperature, we were unable to determine fluorescence lifetimes.

A bathochromic shift of the emission bands is observed in all cases in a frozen matrix (Figure 1b). In **1**, the emission comprises two well‐defined bands at 450 and 550 nm. The high‐energy structureless band at 450 nm is assigned to fluorescence emission from the coumarin fragments, as it is also observed in **3‐BZCA** and **3‐CA** and related complexes [69]. Fluorescence emission from **1** is three times lower than that reported for the non‐bonded chromophore **3‐CA** at 77 K (*φ*_3‐CA_ = 0.01 [84], *φ*_1_ = 0.003 (relative to **3‐CA**)). In the case of the vibronically structured band spanning from 510–720 nm, the long excited state lifetime decay (τ = 30 ms) and large Stokes shift between absorption and emission (Δλ = 222 nm) indicate it corresponds to phosphorescence involving ^3^(π‐π*) coumarin excitation [53], which is further supported computationally (*vide infra*). The long excited‐state lifetime is consistent with a large singlet‐triplet energy gap, usually expected for an organic‐based photosensitizer [53]. As anticipated, the incorporation of heavy atoms (Au and I) into the structure of **1** can be expected to facilitate rapid intersystem crossing, thereby allowing emission from the triplet excited state, a feature that is lacking in **3‐BZCA** and **3‐CA**.

The energy of the triplet excited state in **1** was estimated from the emission spectrum at 77 K (glass) by determining the crossing point of the tangent drawn at the onset of the phosphorescence (Figure S12) [26, 67]. With a triplet energy of *E*_T_ = 56 kcal mol^−1^, **1** could potentially mediate reactions on the triplet surface. This energetic window is comparable to that reported for other common photosensitizers (*fac*‐[Ir(ppy)_3_ *E*_T_ = 58.1 kcal mol^−1^; [Ru(bpy)_3_]^+^;*E_T_* = 49.0 kcal mol^−1^) [26].

While various Au(I) complexes featuring a coumarin moiety exhibit phosphorescence at 77 K [68, 69, 81], their triplet excited state lifetimes are somewhat shorter—in the order of microseconds—than that observed for **1**. Long excited‐state lifetimes are relevant for photocatalytic applications due to the possibility for these excited states to participate in bimolecular events [85]. Thus, we envisioned that **1** could be a candidate for exploring its photoreactivity in triplet EnT reactions (*vide infra*).

With **1** displaying dual emission, we sought to assess the viability of SET events and their relationship to possible competing pathways involving EnT. Using the Rehm‐Weller approximation [86], we estimated the redox potentials in the excited state for **1, [1]** ^−^, **3‐CA**, and **3‐BZCA** to evaluate their potential to mediate SET processes. In the ground state, cyclic voltammetry of **1** exhibits an irreversible anodic peak at *E*_pa_ = 1.03 V versus Fc^+/0^. The irreversibility is retained even at higher scan rates (up to 2 V s^−1^), suggesting that a chemical step follows the electron transfer event. This redox event is observed at a similar potential in **3‐BZCA** (*E*_pa_ = 0.94 V vs. Fc^+/0^), but it is at a less positive potential in **3‐CA** (*E*_pa_ = 0.84 V vs. Fc^+/0^) and is attributed to the irreversible oxidation of the coumarin fragment (Figure S9) [87, 88]. As expected, the functionalization of the pendant amine in **3‐CA** to an amide group in both **1** and **3‐BZCA** renders its oxidation less facile.

With comparable excited state energies (*E*_0,0_ = 3.5–3.6 eV) and oxidation peak potentials in the ground state, the resulting redox potentials in the excited state for **1**, **3‐CA** and **3‐BZCA** (*E_pa_* = −2.54 (**1**), −2.70 (**3‐CA**) and −2.65 **(3‐BZCA)** V vs. Fc^+/0^), indicate these species may act as strong photoreductants compared to other typical photoredox catalysts [24, 86].

We also investigate the effect of deprotonation of the amide group in **1** on its absorption and emission properties, as has been shown to influence the photophysics of photosensitizers bearing proton‐responsive groups [89, 90, 91, 92]. The amidate complex **[1]^−^** was generated in THF solution with an excess of potassium *tert*‐butoxide (Scheme 1). Analysis of the resulting yellow mixture by UV‐vis absorption spectroscopy showed a red shift of the main absorption (λ_max_ = 383 nm) consistent with an increased charge delocalization over the coumarin scaffold (Figure S10). At 77 K, **[1]** ^−^ exhibits only one emission band at 448 nm, in contrast to the two bands observed for the neutral species **1** (Figure 1). Changes in charge delocalization upon deprotonation may alter the energy levels of the orbitals involved in the *S*_1_→*T*_1_ transition, providing efficient non‐radiative deactivation pathways; thus, phosphorescence is not observed from the anionic species. The deprotonation of the NH group on **1** to afford **[1]** ^−^ shifts the oxidation potential to a less positive potential (*E*_pa_ = 0.28 V vs. Fc^+/0^) and lowers the excitation energy (*E*_0,0_ = 3.09 eV) making **[1]** ^−^ a better photoreducing agent than **1**.

## 3 DFT‐Calculations

Density Functional Theory (DFT) calculations were performed to gain a better understanding of the electronic structure of **1** and its influence on its photophysical properties. Geometries were optimized at the TPSS [93]‐D3(BJ) [94, 95]/def2‐TZVP [96]/CPCM [97] (acetonitrile) level of theory, using the crystallographic coordinates of **1** as a starting point. The optimized structure of **1** exhibits similar structural parameters to those determined experimentally (Table S5). Exploration of the electronic structures in the ground and excited states was performed at the CAM‐B3LYP [98]‐B3(BJ) [94, 95]/def2‐TZVP [96]/ CPCM [97] (acetonitrile) level of theory. Inspection of the frontier orbitals indicated that the HOMO of π‐character is centered on the coumarin moiety, whereas the LUMO, of π*‐nature, is mainly localized on the phosphine benzoylamide coumarin fragment (Figure 2). Similarly, in the case of the anionic complex **[1]** ^−^, the frontier orbitals are of π‐character and localized on the coumarin (HOMO) and phosphine benzoyl coumarin (LUMO) scaffold (Figure S28).

Time‐dependent DFT calculations (TD‐DFT) were carried out at CAM‐B3LYP [98]‐B3(BJ) [99]/def2‐TZVP [96]/ CPCM [97] (acetonitrile) level of theory to investigate the nature of the excitations that comprise the low‐energy absorption band. Overall, the simulated spectrum of **1** reasonably resembles the experimental UV‐vis absorption data (Figure S29). The lowest energy absorption band associated with the low‐lying *S*_1_ excited state has the highest oscillator strength (λ_calc_ = 318 nm, λ_exp_ = 328 nm) and originates mainly from a HOMO‐LUMO transition. Analysis of this singlet vertical excitation using Natural Transition Orbitals (NTOs) [100] indicates that it arises from the π‐system of the coumarin and extends in the excited state into the aryl ring of the phosphine backbone, reflecting an intraligand charge transfer (ILCT). This low‐lying *S*_1_ state lies 90.0 kcal mol^−1^ above the *S0* ground state. Additionally, we optimized the structure of **1** in the triplet state to gain insight into its electronic nature. Notably, the triplet state in the fully relaxed geometry lies 55.5 kcal mol^−1^ above *S*_0_, agreeing with our experimentally estimated value (*vide supra*), and 34.5 kcal mol^−1^ below *S*_1_. Hence, emission from the triplet state is energetically accessible (Figure 2b). The spin density plot for *T*_1_ indicates unpaired electron density at the benzoylcoumarin fragment (Figure 2c). Overall, these results align with our experimental observations, demonstrating that the emission from complex **1** is localized around the coumarin scaffold.

## 4 Photocatalysis

The ability of **1** to access reactivity from a triplet excited state was explored here in the arylative cyclization of *o*‐alkynyl phenols with diazonium salts. This reaction has been proposed earlier to involve low‐lying triplet excited states and potentially proceed via EnT [34, 50, 101]. We used similar experimental conditions [34] to test the effect of irradiation on the reaction of *p*‐nitrophenyldiazonium tetrafluoroborate (*A^NO2^*) with 2‐(4‐tolylethynyl)phenol (*B*) in the presence of **1** (5 mol%). Irradiation at 365 nm does not improve product yields, ascribed to direct photolysis of the aryl diazonium salts [102]. Hence, although **1** absorbs at 370 nm, we chose to irradiate at 450 nm to avoid this side reaction.

Comparable yields for the cross‐coupling product 3‐(4‐Nitrophenyl)‐2‐(*p*‐tolyl)benzofuran (*C*) are obtained when either complex **1** or Ph_3_PAuCl (in the absence of photosensitizer) is used as a photocatalysts (**1** = 51%, Ph_3_PAuCl = 42%, see Table 2, entries 1 and 4). These outcomes indicate that the coumarin scaffold in **1** has a poor influence on product formation. Also, they align with our previous findings for this reaction [34], in which Ph_3_PAuCl affords *C* without the need for an added photocatalyst. Notably, improvement is not observed upon adding **3‐CA** (5 mol%), as *C* is formed in 36% yield (Table 2, entry 5). Of note, the addition of **3‐BZCA** inhibits product formation, with the yields for the cross‐coupling product decreasing significantly (7% for C; see Table 2, entry 6).

**TABLE 2:** Yields (determined by NMR spectroscopy) for the arylative cyclization of *p*‐nitrophenyl diazonium tetrafluoroborate (*A^NO2^*) with 2‐(4‐tolylethynyl)phenol (*B*).^a^

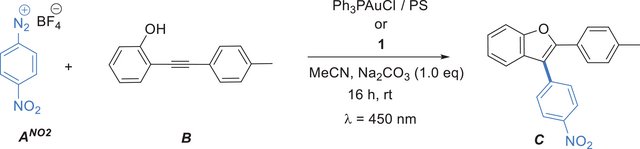		
Entry	Conditions	Yield (%)^b^ of *C*
1	**1** (5 mol%)	51
2	**1** (5 mol%)	48^c^, ^d^
3	**1** (5 mol%) in the dark	12^d^
4	Ph_3_PAuCl (5 mol%)	42 (32)^e^
5	Ph_3_PAuCl (5 mol%), **3‐CA** (5 mol%)	36
6	Ph_3_PAuCl (5 mol%), **3‐BZCA** (5 mol%)	7^d^

^a^ Reactions were carried out in duplicate at rt, irradiating at 450 nm (Blue LED, 115 mW optical power).

^b^ Average yields by ^1^H NMR spectroscopy using 3,5‐dinitro‐methylbenzoate as internal standard.

^c^ Experiment recorded under irradiation at 365 nm.

^d^ Single experiment.

^e^ Reported yield [34.].

We excluded the possibility that the cross‐coupling reaction occurs via photothermal activation, as a significant temperature increase was not observed during irradiation (Figure S26). Moreover, product formation greatly benefits from irradiation, as seen in entries 1 and 3.

We wondered if the poor performance of **1** may be due to the emergence of competing pathways since it can, in principle, participate in SET and EnT reactions. A comparison between the redox potentials of **1** (in the excited state, *E*_ox_*(**1** ^•+^/1*) = −2.16 V vs. SCE) and the previously reported value for the phenyl diazonium cation (−0.1 vs. SCE) [103] indicates SET is thermodynamically feasible under the reaction conditions. Therefore, we carried out quenching experiments in solvents of varying polarity to determine whether competing SET pathways are present by monitoring changes in the emission spectrum of **1** upon the addition of either *A^NO2^* or *B* as quenchers at 298 K. While **1** emits weakly under these conditions, a trend was found. *A^NO2^* quenches the emission of **1** in both MeCN (K_SV_ [mM^−1^] = 5.49, Figure S17) and CH_2_Cl_2_ (K_SV_ [mM^−1^] = 2.14), suggesting that the photoactivation pathway may proceed via SET (with **1** being oxidatively quenched by the diazonium salt, due to the favorable redox potentials), which also aligns with the slight variation in K_SV_ with solvent polarity. In the case of *B*, we detected quenching only in CH_2_Cl_2_ (K_SV_ [mM^−1^] = 1.32, Figure S19a), whereas in MeCN the strong fluorescence of *B* overlaps with the emission of **1** (Figure S19b). A possible origin of this observation could be the coordination of the unsaturated substrate *B* to the gold center, leading to the formation of the corresponding vinyl gold(I) intermediate, from which EnT can take place [34, 50].

We also explored the activity of **1** as an EnT catalyst in the E/Z photoisomerization of dimethyl fumarate [67]. However, we did not detect the formation of the corresponding *cis*‐isomer (dimethyl maleate) upon irradiation of a mixture of dimethyl fumarate and **1** (10 mol%) at 365 nm (Figure S27).

Thus, given the potential of **1** to mediate SET reactions, we turned our attention to investigating the light‐driven diarylation of diazonium salts [104] with phenylboronic acid (D). This reaction, previously investigated by Fouquet [105] and Lee [106], was proposed to occur via SET. We explored the effect of light on the reactivity of **1** in the presence of *A* ^NO2^ and *D*, using conditions similar to those reported by Fouquet. Although **1** affords the cross‐coupling product 4‐nitro‐1,1′‐biphenyl (*F* ^NO2^) in the dark (34% yield, see Table 3, entry 2), irradiation of the mixture at 450 nm results in higher yields for *F* ^NO2^ (63%). We ruled out photothermal activation, as a substantial increase in temperature was not observed during the photoreaction (Figure S26).

**TABLE 3:** NMR yield for the C(sp^2^)‐C(sp^2^) cross‐coupling of aryldiazonium salts (*A^R^*) with phenyl boronic acid (*D*).^a^

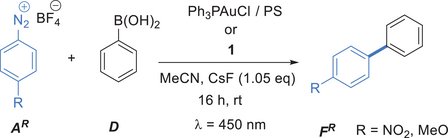			
		Yield (%)^b^ of *F^R^*	
Entry	Conditions	R = NO_2_	R = MeO
1	**1** (10 mol%)	63	35
2	**1** (10 mol%) in the dark	34^c^	4
3	Ph_3_PAuCl (10 mol%), **3‐BZCA** (10 mol%)	19	34
4	Ph_3_PAuCl (10 mol%), **3‐CA** (10 mol%)	43	34
5	Ph_3_PAuCl (10 mol%), **3‐CA** (4 mol%)	43	47
6	Ph_3_PAuCl (10 mol%), Acridinium (4 mol%)	35 (76)^d^	49 (55)^d^
7	Ph_3_PAuCl (10 mol%), [Ru(bpy)_3_]^2+^ (2 mol%)	38 (75)^d^	48 (63)^d^
8	Ph_3_PAuCl (10 mol%)	34	40

^a^ Reactions were carried out at rt in duplicate under irradiation at 450 nm (Blue LED, 115 mW optical power).

^b^ NMR average yields of two experiments calculated by ^1^H NMR using 3,5‐dinitro‐methylbenzoate as internal standard.

^c^ Single experiment, ^1^H NMR yield using tetrachloroethane.

^d^ Previously reported isolated yield [105].

The activity of the free chromophores **3‐CA/3‐BZCA** in the presence of Ph_3_PAuCl (1:1 ratio) was determined to evaluate the impact of the covalently bound coumarin in **1** on catalytic performance. When **3‐CA** is used, *F* ^NO2^ is afforded in 43% yield. Lowering the concentration of **3‐CA** does not affect catalytic performance, as similar yields were obtained across catalyst loads (see Table 3, entries 4 and 5).

When **3‐BZCA** is used as a photosensitizer, lower yields for *F^NO2^* were obtained (see entry 3 in Table 3). We discard that this could be caused by the presence of the amide group in **3‐BZCA**, as with **1,** *F* ^NO2^ is obtained in higher yields (entries 1 and 3, Table 3). While there is a slight improvement, only a little benefit is realized from merging the chromophore with the Au(I) catalyst when using *A^NO2^*.

Comparable yields were obtained for **1** or Ph_3_PAuCl in combination with **3‐CA**/**3‐BZCA** when *p*‐methoxyphenyl diazonium tetrafluoroborate (*A^MeO^*) is used as substrate. In all cases, 4‐methoxy‐1,1’‐biphenyl (*F^MeO^*) is yielded in 34%–35% (entries 1, 3, and 4, Table 3). Fouquet reported similar conclusions for this reaction, in which electron‐rich diazonium salts showed lower conversion [105].

The activity of [Ru(bpy)_3_][PF_6_]_2_ and 9‐mesityl‐10‐methylacridinium in combination with Ph_3_PAuCl—as reported by Fouquet [105]—was evaluated with the present experimental setup. While the yields obtained here are slightly lower, we find that in our setup, **3‐CA** can compete with common photocatalysts in gold catalysis (Table 3, entries 5–7). Notably, **3‐CA** exhibits a high redox potential in the excited state (E*_ox_ (**3‐CA^+•^**/**3‐CA** ^*^) = −2.70 V vs. Fc^+/0^, −2.32 vs. SCE) [107], which is substantially cathodically shifted compared to that reported for [Ru(bpy)_3_][PF_6_]_2_ (−0.81 V vs. SCE) [108]. Interestingly, Ph_3_PAuCl performed comparatively well in the absence of an external photoredox catalyst for both substrates in our setup.

Stern–Volmer quenching experiments using **1** as the catalyst with either *A^NO2^* or *D* were performed at 298 K, showing that only A*^NO2^* quenches the emission (K_SV_ = 5.49 mM^−1^, see Figures S17 and S18), which is consistent with previous proposals for gold complexes with covalently attached photosensitisers [58]. Given the excited state redox potentials of **1**, we attribute this observation to oxidative quenching in the presence of *A^NO2^*, likely following an SET mechanism due to thermodynamically favored electron transfer (*vide supra*).

## 5 Conclusions

In summary, we herein explore the light‐driven reactivity of a new Au(I) phosphine complex **1** with a covalently attached 3‐amino coumarin chromophore. The photocatalytic activity of **1** was evaluated in two representative cross‐coupling reactions, which are postulated to proceed via either energy or electron transfer. Our observations indicate that covalent attachment of the coumarin provides modest benefit to photocatalytic performance, as the separate chromophore and gold catalyst yield similar results. Although **1** can undergo both electron and energy transfer events—as it exhibits fluorescence and phosphorescence at 77 K—intramolecular photosensitization may provide alternative non‐productive (deactivation) pathways (e.g., nonradiative decay) that may interfere with the photoactivation.

In our case, this strategy provided limited improvements in the arylative cyclization of *o*‐phenols and in diarylation reactions, with an overall 15%–20% increase in yield compared to the separate catalysts. Bräse, in contrast, reported a decrease in 7% yield for the Meyer–Schuster rearrangement for the bifunctional catalyst (a) (Chart 1) [56], while Breher reported a 45% improvement for the catalyst (b) (Chart 1) in the *P*‐arylation of diazonium salts [60], both compared to the uncoupled units. These observations indicate that a modest—or non—benefit can be obtained with bifunctional catalysts comprising a photosensitizer and gold catalyst, raising awareness of their practical benefits in photocatalytic applications. While these bifunctional systems hold potential to drive light‐induced events, a deeper understanding of the factors governing efficient photosensitization is necessary to enhance and leverage the reactivity of excited states in gold photocatalysis.

## Conflicts of Interest

The authors declare no conflicts of interest.

## Supporting information




**Supporting File**: The authors have cited additional references within the supporting information [109, 110, 111, 112, 113, 114, 115, 116, 117, 118, 119, 120, 121, 122, 123, 124, 125, 126, 127, 128].

## Data Availability

The data that support the findings of this study are openly available in dataverse.nl at https://doi.org/10.34894/NKJQNZ.

## Endnote

1 For an alternative method for preparing gold(I) phosphane amide complexes see: Kriechbaum, M.; List, M.; Himmelsbach, M.; Redhammer, G. J.; Monkowius, U. Peptide coupling between amino acids and the carboxylic acid of a functionalized chlorido‐gold(I)‐phosphane. *Inorg. Chem*. **2014**, *53* (19), 10602‐10610.
