# Supplementary material for: Photosensitizing Au(I) Catalysis With Coumarins
Source: Chemistry. 2026 Jun 25;32(38):e71185. doi: 10.1002/chem.71185 (PMC13660497; doi:10.1002/chem.71185)
Supplement: Supplementary file 1 [file CHEM-32-e71185-s001.pdf]

Supporting information

Photosensitizing Au(I) catalysis with coumarins

Liliana, Capulin Flores,[a] Jacob, J. van der Wal,[a] and Johannes E. M. N. Klein.[a*]

[a]Stratingh Institute for Chemistry, University of Groningen, Nijenborgh 3, 9747 AG Groningen, The Netherlands.
Email: j.e.m.n.klein@rug.nl

Table of contents

	Page
General considerations	S3
Synthesis of 3-aminocoumarin	S3
Synthesis of 3-benzoylamide coumarin	S4
Synthesis of (*p*-COOH-Ph)Ph_2PAu-Cl	S4
Synthesis of complex **1**	S4
NMR Spectroscopy	S5
FT-IR Spectroscopy	S8
High-resolution mass spectrometry	S9
X-ray diffraction	S10
Cyclic voltammetry	S11
Photophysical measurements	S12
Quenching experiments	S16
Lifetime measurements	S19
Photocatalysis	S20
DFT calculations	S27
References	S31

General considerations

All reactions, except ligand synthesis, were performed under an atmosphere of N_2 using conventional Schlenk and vacuum-line techniques. CH_2Cl_2 was dried over calcium hydride, degassed with five freeze-pump-thaw cycles and vacuum transferred prior to use. Ethanol was dried over molecular sieves (4Å). Anhydrous acetonitrile was used as received (Sigma Aldrich, 99.8%). $(Me)_2S$ (Janssen Chimica, 99%), (*p*-COOH-Ph)Ph_2P (Sigma-Aldrich, 97%), salicyladehyde (TCI, 98%), L-proline (TCI, 99%), ethylnitroacetate (TCI, >95%), stannous chloride dihydrate ($SnCl_2$•$2H_2O$) (Sigma-Aldrich, 98%), 2-chloro-1-methylpyridinium iodide (Sigma-Aldrich, 97%), phenylboronic acid (Sigma-Aldrich, 97%), *p*-nitrophenyldiazonium tetrafluoroborate (TCI, >98%), *p*-methoxyphenyldiazonium tetrafluoroborate (TCI, >98%) ferrocene (TCI, >98%) and tetrabuthylammonium hexafluorophosphate (TCI, >98%) were used as received. $(Me)_2$SAu-Cl,[1] (*p*-COOH-Ph)Ph_2PAu-Cl,[2] 3-amino-coumarin[3] and 2-(4-tolylethynyl)phenol[4] were prepared following previously reported procedures. $CDCl_3$ (Eurisotop, 99.8 atom %D) was passed through basic aluminium oxide prior use.

NMR spectra were recorded on Mercury 400, Varian Inova 500 or Bruker 600 MHz spectrometers. Residual solvent signals were used as internal reference for ^{1}H and $^{13}C\{^{1}H\}$ spectra and reported in ppm relative to TMS (0 ppm). Complete assignments were aided by two-dimensional NMR experiments (COSY, HSQC, HMBC) using standard pulse sequences. FT-IR spectra were collected in solid state on a JASCO 4700 series FT-IR spectrophotometer in transmission mode. HRMS was performed on a Thermo Scientific Orbitrap Exploris 480, with ESI source. UV-vis spectra were recorded on an Agilent Technologies 8453 UV-vis spectrophotometer. Cyclic voltammetry experiments were conducted using a hand-held CH1200B® potentiostat. Fluorescence spectroscopy was measured on a spectrofluorometer FS5 from Edinburgh instruments® equipped with ozone-free Xenon arc lamp and a single photon counting. X-ray diffraction data were collected at 100 K on a Bruker D8 Venture diffractometer with Mo Kα (λ= 0.71073 Å) radiation source. Crystal structures were refined using the SHELXL [5]software. Non-hydrogen atoms were refined anisotropically.

Synthesis of 3-aminocoumarin

Based on previously reported procedure.[3] In a round-bottom flask, 0.55 mL of salicyladehyde (0.640 g, 5.2 mmol) and 0.5 mL of ethylnitroacetate (5.2 mmol, 0.700 g) were dissolved in 15 mL of dry ethanol. Then, 0.181 g (1.6 mmol) of L-proline was added, observing that the solution turned yellow. The mixture was stirred for 4 h and the solvent evaporated to dryness. The mixture was redissolved in 60 mL of $CHCl_3$ and extracted with water (3 x 50 mL). Lastly, the organic phase was washed with brine (50 mL) and dried over $MgSO_4$. The crude was purified by column chromatography using silica gel as stationary phase and CH_2Cl_2 as eluent yielding 293 mg (29%) of 3-nitrocoumarin. A suspension of 3-nitrocoumarin (0.191 g, 1 mmol) was prepared in 8 mL of concentrated HCl (35 %). Stannous chloride ($SnCl_2$•$2H_2O$) was added in small portions (1.7 g, 7.5 mmol) and the reaction stirred for 5 h. The round-bottom flask was cooled down in an ice bath and 20 g of ice were added to the reaction mixture. A solution of NaOH (5 M) was added dropwise until pH = 10. The suspension was extracted with ethyl acetate (3 x 25 mL). The organic phase was dried over $MgSO_4$, and the solvent was removed under reduced pressure. The crude was recrystallized from hot MeOH (3 mL), affording pale-yellow crystals (0.090 g, 56 %). ^{1}H NMR ($CDCl_3$, 25 °C, 400 MHz) δ/ppm: 4.25 (s, 2H, NH_2-coumarin), 6.70 (s, 1H, 4-CH coumarin) 7.21-7.31 (m, 4 H, 6-CH, 7-CH, 8-CH, 9-CH coumarin). $^{13}C\{^{1}H\}$ NMR ($CDCl_3$, 25 °C, 150 MHz) δ/ppm: 110.9 (4-CH coumarin), 116.2 (9-CH coumarin), 121.2 (5-C coumarin), 124.6 (8-CH coumarin), 125.1 (7-CH coumarin), 126.6 (6-CH coumarin), 132.0 (10-C coumarin), 149.1 (3-C coumarin), 159.5 (-C(=O)- coumarin). HRMS (ESI+) m/z: Calcd. for $[C_9H_7NO_2H]^+$ = 162.05496. Found = 162.0597. (ESI-) m/z: Calcd. for $[C_9H_7NO_2H]^-$ = 161.04376. Found = 161.02439. Spectroscopic data matched the previously reported.[3b] UV-vis (25 °C, MeCN), λ_{max}/nm = 3233 nm (ε = 54 000 $M^{-1}cm^{-1}$).

Synthesis of 3-benzoylamide coumarin

Following a previously reported procedure,[6] a N_2-filled two-necked round-bottom flask was charged with 0.128 g (0.79 mmol, 1 eq.) 3-amino coumarin which were dissolved in 7 mL of anhydrous CH_2Cl_2. Then, 0.07 mL (0.86 mmol, 1.1 eq.) of pyridine were added, and the reaction mixture was cooled to 0 °C. 0.10 mL (0.86 mmol, 1.1 eq.) of benzoyl chloride were added dropwise, the ice bath was removed, and the reaction mixture was stirred overnight at room temperature. The solvent was evaporated to dryness and the residue purified by column chromatography using silica gel as stationary phase and a mixture of pentane/ethyl acetate (90:10) as eluent (R_f = 0.59). Upon evaporation, white crystals were obtained (0.162 g, 77%). The product was stored under an atmosphere N_2 due to the hygroscopic nature of the compound. ^{1}H NMR ($CDCl_3$, 25 °C, 400 MHz) δ/ppm: 7.29-7.33 (m, 2H, 6-CH, 7-CH, coumarin), 7.43-760 (m, 5 H, 5-CH, 8-CH coumarin, *m*-CH, *p*-CH Ph-(C=O)-NH), 7.93 (d 2 H, ^3J = 8.2 Hz, *o*-CH Ph-(C=O)-NH), 8.83 (s, 1H, NH –(C=O)-NH), 8.85 (s, 1H, 4-CH coumarin). The spectroscopic data match the previously reported.[7] UV-vis (25 C, MeCN), λ_{max}/nm = 323 nm (ε = 45 808 $M^{-1}cm^{-1}$).

Synthesis of (*p*-COOH-Ph)Ph_2PAu-Cl

Prepared as previously reported by Monkowius and co-workers.[2a] 0.202 g (0.68 mmol) of $(Me)_2$SAu-Cl and 0.210 g (0.68 mmol) were added in N_2-filled Schlenk and dissolved in 8 mL of dry DCM. The reaction vessel was covered with aluminum foil and stirred for 1 h at room temperature. The volume of the solution was reduced to half and the compound was precipitated by addition of pentane. (0.223 g, 56%). ^{1}H NMR ($CDCl_3$, 25 °C, 400 MHz) δ/ppm: 7.49-7.64 (m, 12 H, *o*-CH(Ph), *m*-CH(Ph), *p*-CH(Ph), *o*-CH (*p*-COOHPh)), 8.17 (d, ^{3}J = 8.5 Hz, m-CH (*p*-COOHPh)). ^{31}P{^{1}H] ($CDCl_3$, 25°C, 100 MHz) δ/ppm: 33.15. Matches previously reported spectroscopic data.[2a]

Synthesis of Complex 1

A Schlenk flask, equipped with a stirring bar and covered with aluminum foil, was charged with 0.103 g (1 eq., 0.19 mmol) of (*p*-COOH-Ph)Ph_2PAu-Cl and 0.078 g of 2-chloro-1-methylpyridinium iodide (1.5 eq., 0.31 mmol) under an atmosphere of N_2. The solids were dissolved in 1 mL of anhydrous CH_2Cl_2 and 0.13 mL of NEt_3 (5 eq. 0.93 mmol). The mixture was stirred for 1 h and then, 0.034 g (1 eq., 0.19 mmol) of 3-aminocoumarin were added. The reaction was heated to 30 °C and stirred for 24 h. Subsequently, the solvent was removed under reduced pressure and the yellow crude purified by column chromatography on silica gel using CH_2Cl_2 as the eluent (R_f = 0.71). Upon evaporation, a white powder was obtained, which was recrystallized by slow diffusion of pentane into a CH_2Cl_2 solution. (0.130 g, 65%). ^{1}H NMR ($CDCl_3$, 25 °C, 600 MHz) δ/ppm: 7.33-7.37 (m, 2 H, 6-CH, 7-CH, coumarin), 7.48-760 (m, 12 H, Ph (PPh_2); 5-CH, 8-CH coumarin), 7.68 (dd 2 H, $^3J_{P\text{-}H}$= 12.6 Hz, ^3J = 8.5 Hz, *o*-CH Ph_2P-Ph-(C=O)-NH), 7.99 (dd 2 H, $^4J_{P\text{-}H}$= 2.0 Hz, ^3J = 8.5 Hz, *m*-CH Ph_2P-Ph-(C=O)-NH), 8.83 (s, 1H, NH –(C=O)-NH), 8.85 (s, 1H, 4-CH coumarin). ^{13}C{^{1}H} NMR ($CDCl_3$, 25 °C, 150 MHz) δ/ppm: 116.5 (9-CH coumarin), 119.6 (6-C coumarin), 123.8 (5-C coumarin), 124.1 (4-CH coumarin), 125.4 (7-CH coumarin), 127.7 (d $^3J_{C\text{-}P}$= 12.7 Hz, *m*-CH Ph_2P-Ph-(C=O)-NH), 128.0 (8-CH coumarin), 128.4 (10-C coumarin), 129.5 (d $^3J_{C\text{-}P}$= 11.9 Hz, *m*-CH PPh_2), 130.2 (*p*-CH PPh_2), 132.3 (*ipso*-C PPh_2), 134.2 (d $^2J_{C\text{-}P}$= 14.4 Hz, *o*-CH PPh_2), 134.4 (d $^2J_{C\text{-}P}$= 14.4 Hz, *o*-CH Ph_2P-Ph-(C=O)-NH), 134.8 (*p*-C Ph_2P-Ph-(C=O)-NH), 136.5 (*ipso*-C Ph_2P-Ph-(C=O)-NH), 150.0 (3-C coumarin), 158.9(-C(=O)- coumarin), 164.8 (-(C=O)- amide). ^{31}P{^{1}H] ($CDCl_3$, 25°C, 150 MHz) δ/ppm: 38.63. FT-IR(ATR) ν_{max}/cm^{-1}: 1705(s) (C(=O) coumarin), 1663 (s) (C=(O) amide). UV-vis (25 C, MeCN), λ_{max}/nm = 328 nm (ε = 29 450 $M^{-1}cm^{-1}$). HRMS (ESI+) m/z: Calcd. for $[C_{28}H_{20}AuINO_3PNa]^+$ = 796.98169. Found = 796.98070. (ESI-) m/z: Calcd. for $[C_{28}H_{19}AuINO_3P]^-$ = 771.9816. Found = 771.9818.

Synthesis of complex [1]$^-$

This complex was generated *in situ* from a solution of **1** in dry THF. Inside the glovebox, 9.7 mg (0.013 mmol) of complex **1** were dissolved in 2 mL of THF, then 5 equivalents (1.5 mg, 0.07 mmol) of KOtBu were added. Upon stirring, the solution turned bright yellow. The mixture was filtered to remove the excess of KOtBu. Analysis of the reaction mixture by NMR and UV-vis indicated successful deprotonation of the amide group. ^{1}H NMR (THF-d_8, 25 °C, 400MHz) δ/ppm: 7.10-7.12 (m, 3 H, 6-CH, 7-CH, and 9-CH coumarin), 7.32 (d, 1 H, 8-CH, coumarin), 7.49-7.64 (m, 12 H, Ph (PPh$_2$), *m*-CH Ph$_2$P-Ph-(C=O)-N$^-$), 7.68 (dd 2 H, $^3J_{P-H}$= 12.6 Hz, ^3J = 8.5 Hz, *o*-CH Ph$_2$P-Ph-(C=O)-N$^-$), 8.18 (dd 2 H, $^4J_{P-H}$= 2.4 Hz, ^3J = 8.3 Hz, *m*-CH Ph$_2$P-Ph-(C=O)-N), 8.64 (s, 1H, 4-CH coumarin). ^{31}P{^{1}H] (THF-d_8, 25°C, 100 MHz) δ/ppm: 40.10.

NMR Spectroscopy

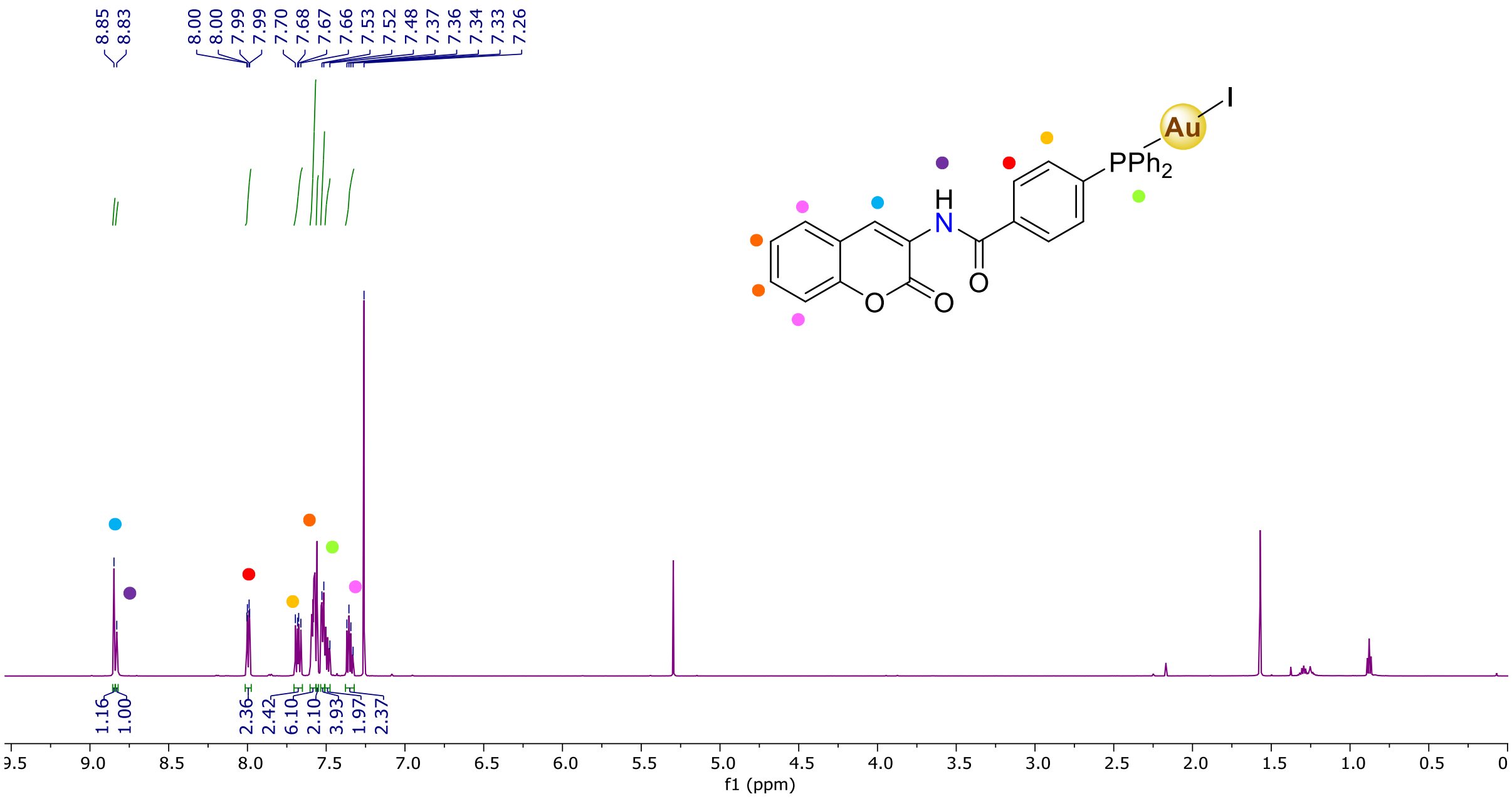

Figure S1. ^{1}H NMR spectrum of complex **1** in $CDCl_3$ at rt.

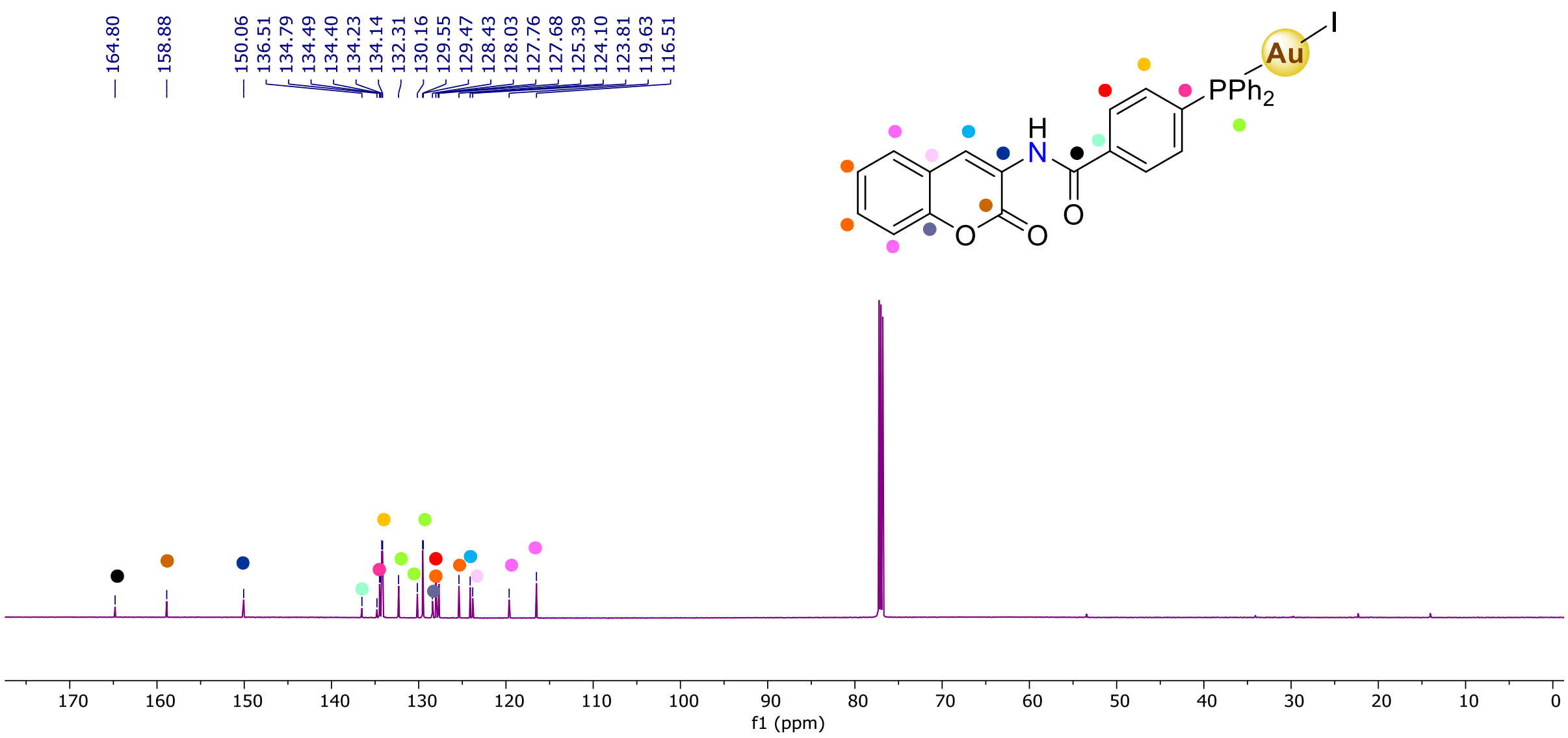

Figure S2. ^{13}C{^{1}H] NMR spectrum of complex **1** in $CDCl_3$ at rt.

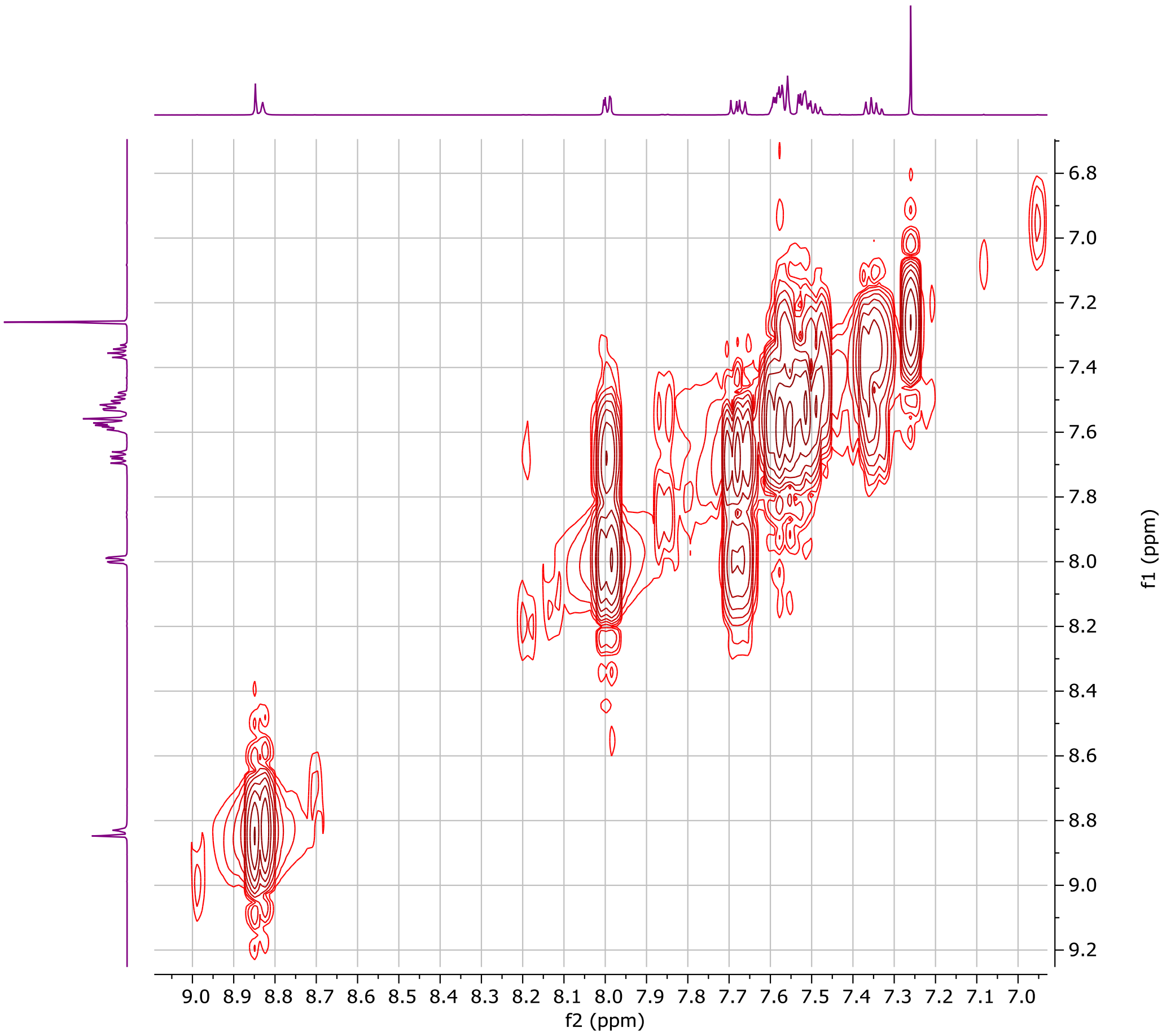

Figure S3. COSY experiment of complex **1** in $CDCl_3$ at rt.

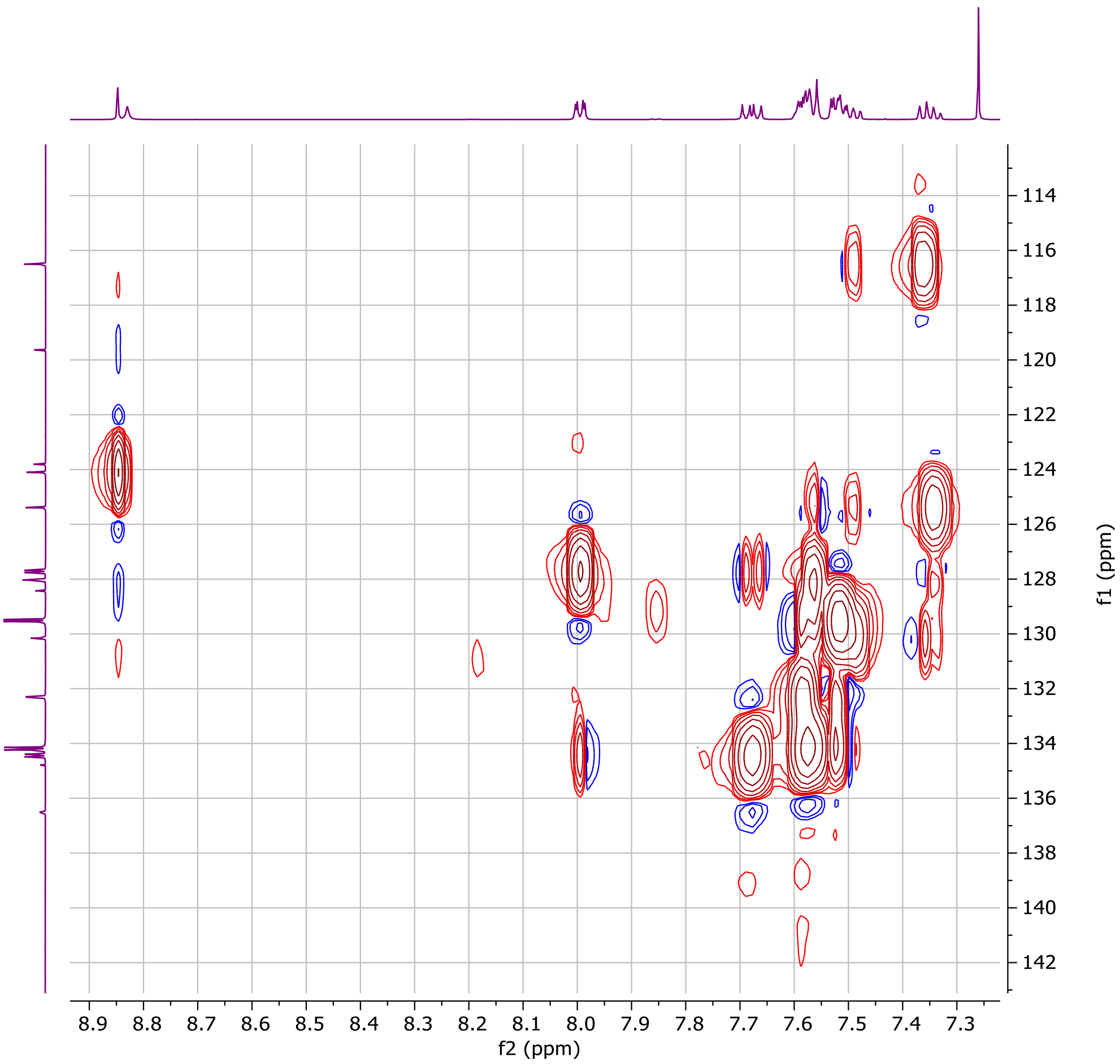

Figure S4. HSQC experiment of complex **1** in $CDCl_3$ at rt.

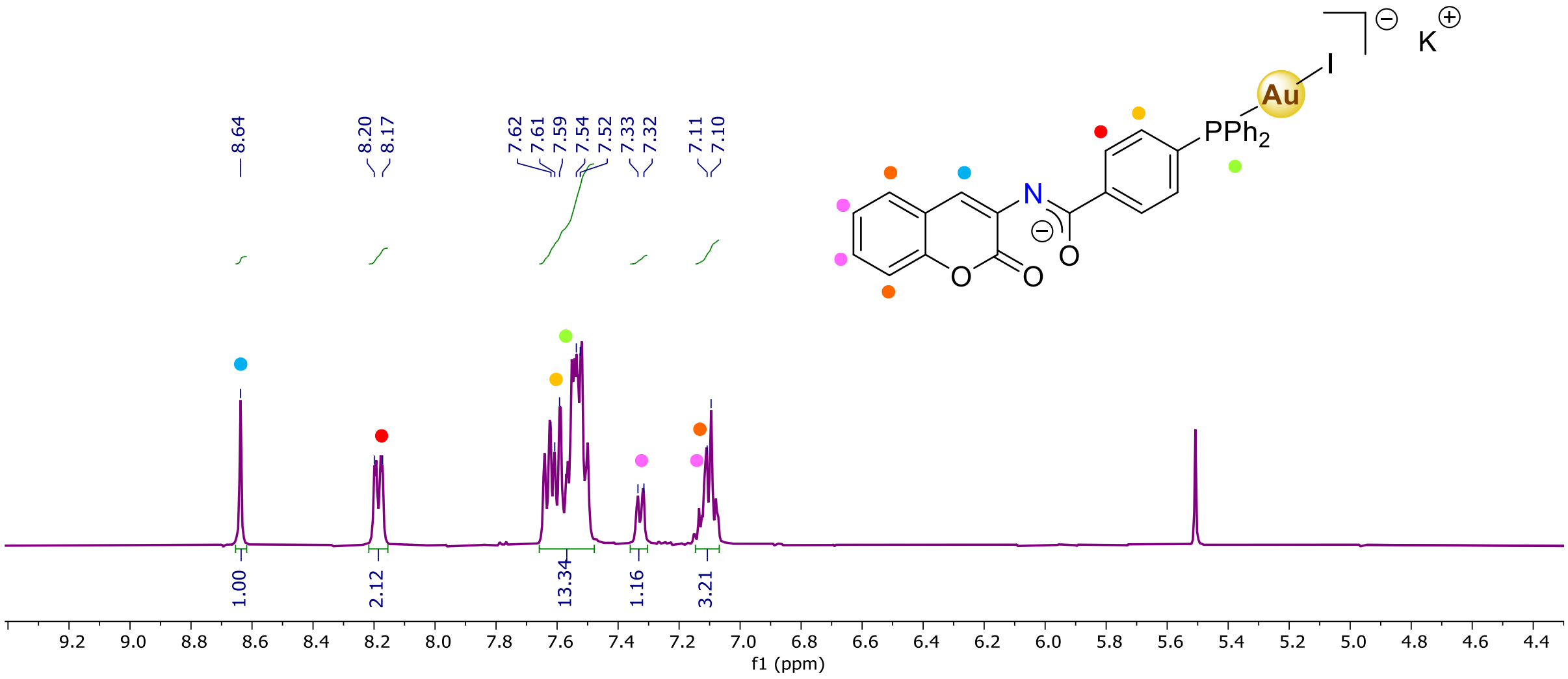

Figure S5. ^{1}H NMR spectrum of complex **[1]**$^{-}$ in THF-d_8 at rt.

FT-IR Spectroscopy

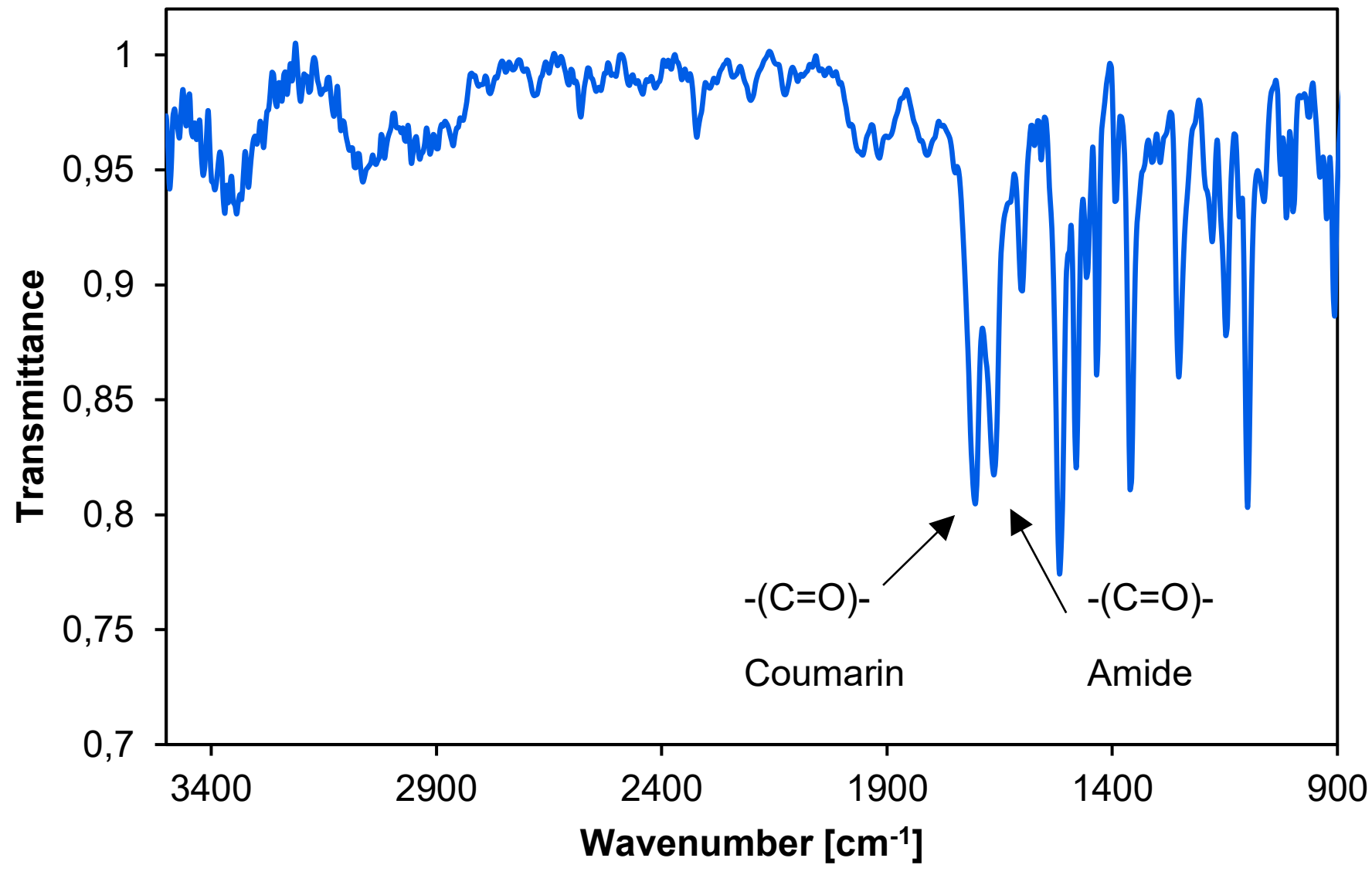

Figure S6. FT-IR spectrum of complex **1**.

High resolution mass spectrometry

a)

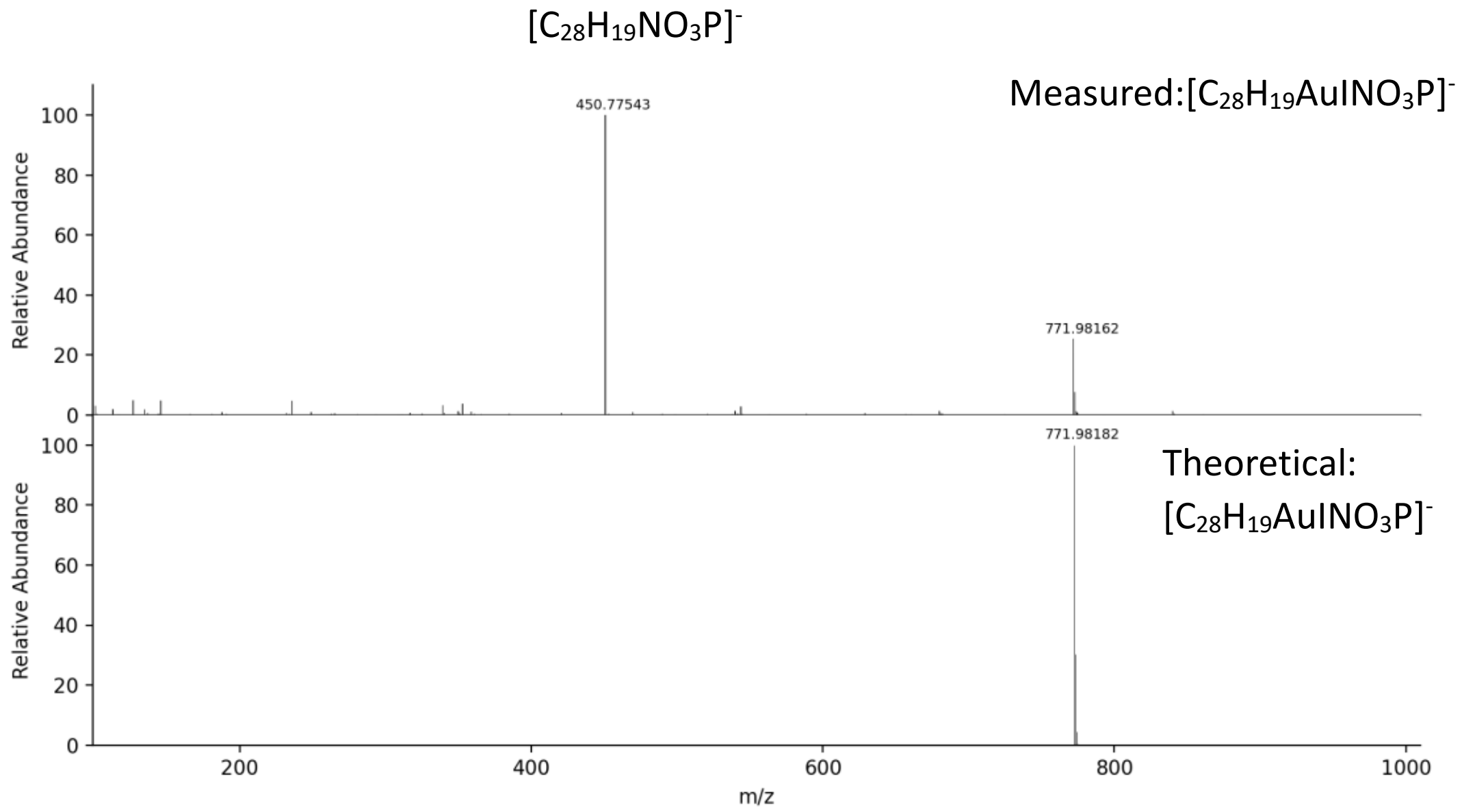

b)

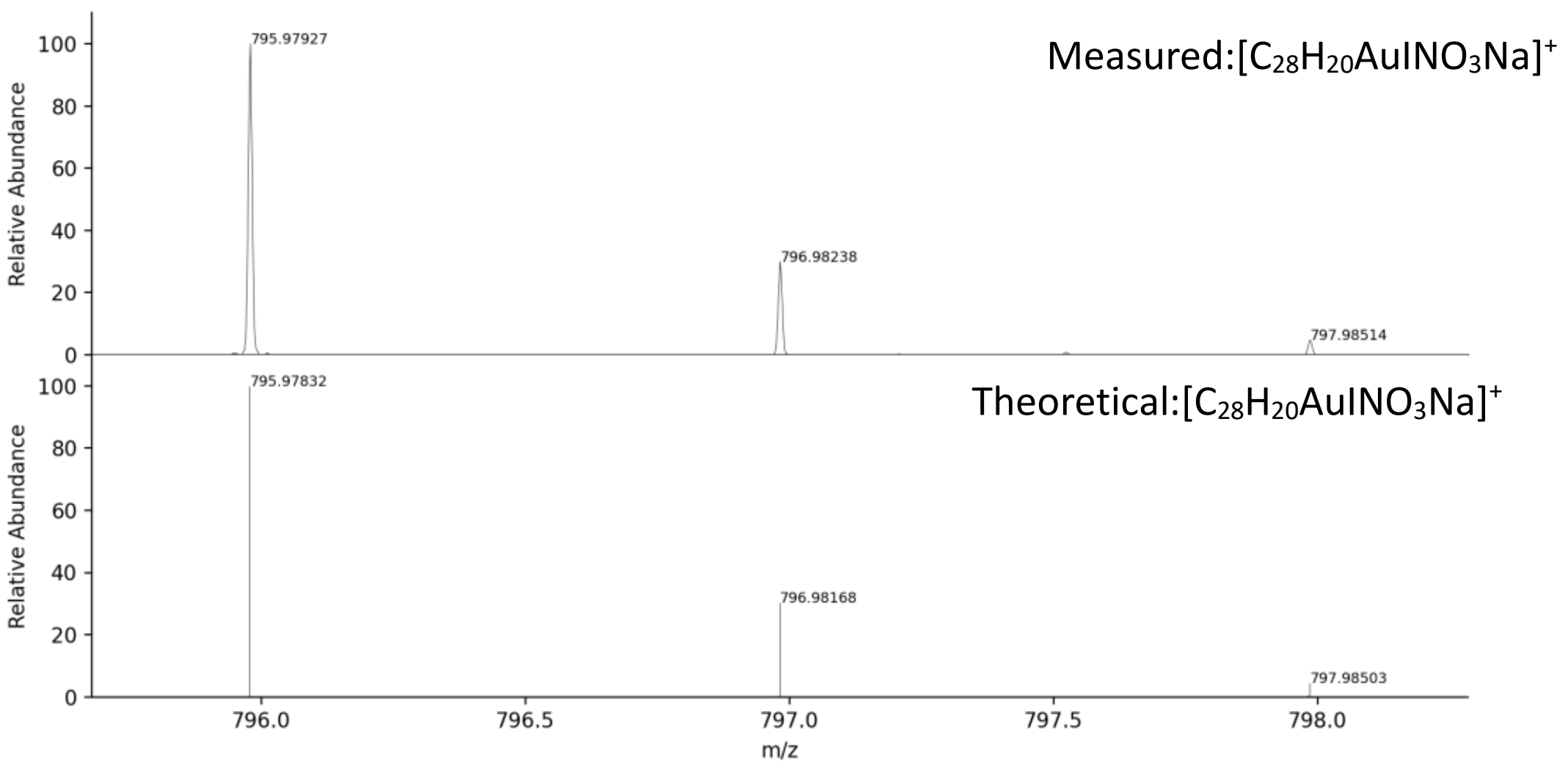

Figure S7. HRMS, ESI a) negative and b) positive mode of complex **1.**

X-ray diffraction

Crystals suitable for X-ray diffraction were collected by slow diffusion of pentane into a CH_2Cl_2 solution of **1**. The data were collected at 100 K using a Bruker D8 Venture diffractometer. Data collection and reduction were made using the Bruker software suite APEX4. A multiscan absorption correction was applied based on the intensities of symmetry-related reflections measured at different angular settings (SADABS).[8] The structures were solved by direct methods using SHELXT,[9] and refinement of the structure was performed using SHELXL.[5] Hydrogen atoms were placed in geometrically calculated positions and allowed to ride on their carrier atoms with an isotropic displacement parameter related to the equivalent displacement parameter of the corresponding carrier atom. Crystal data and details of their collection and refinement are shown in Table S1. Refinement of the molecular structure of complex **1** indicates the presence of one additional solvent molecule in a highly disordered fashion. Solvent contribution was subtracted using PLATON/SQUEEZE routine.[10]

Table S1. Crystallographic data for complex 1.

**Chem. Formula**	$C_{28}H_{20}AuINO_3P$
**Complex**	1
**Mr**	773.28
**Cryst syst**	Triclinic
**Color, habit**	Colorless, needle
**Size (mm)**	0.25 x 0.20 x 0.02
**Space group**	*P*-1
**a (Å)**	9.1326(5)
**b (Å)**	9.7272(5)
**c (Å)**	16.4060(09)
**α (°)**	81.322(2)
**β (°)**	88.803(2)
**γ (°)**	69.745(2)
**V (Å^3)**	1350.87(13)
**Z**	2
**ρ_{calc}, g/cm^3**	1.901
**Radiation (Å)**	Mo, Kα, 0.71073
***μ* (Cu Kα), mm^{-1}**	6.678
***F*(000)**	732
**Temp (K)**	100(2)
**θ range (°)**	2.75-26.46
**Data collected (*h*,*k*,*l*)**	-11:11; -12:12; -20:20
**No. of rflns collected**	9963
**No. of indpndt reflns**	5299
**Observed reflns $F_0 \geq 2\sigma (F_0)$**	5260
**R(F) %**	3.22

Cyclic voltammetry

The voltammograms were recorded under an atmosphere of N_2 at 100 mV/s using a three-electrode set-up with a glassy carbon working electrode, a Ag/AgCl pseudo reference electrode, and a Pt wire as a counter electrode. The glassy carbon electrode was polished with alumina oxide and sonicated with DI water and acetone prior to use. Measurements were performed in MeCN (unless otherwise stated) using NBu_4PF_6 as a supporting electrolyte in 0.1 M concentration and internally referenced with ferrocene, with an analyte concentration of 1 mM.

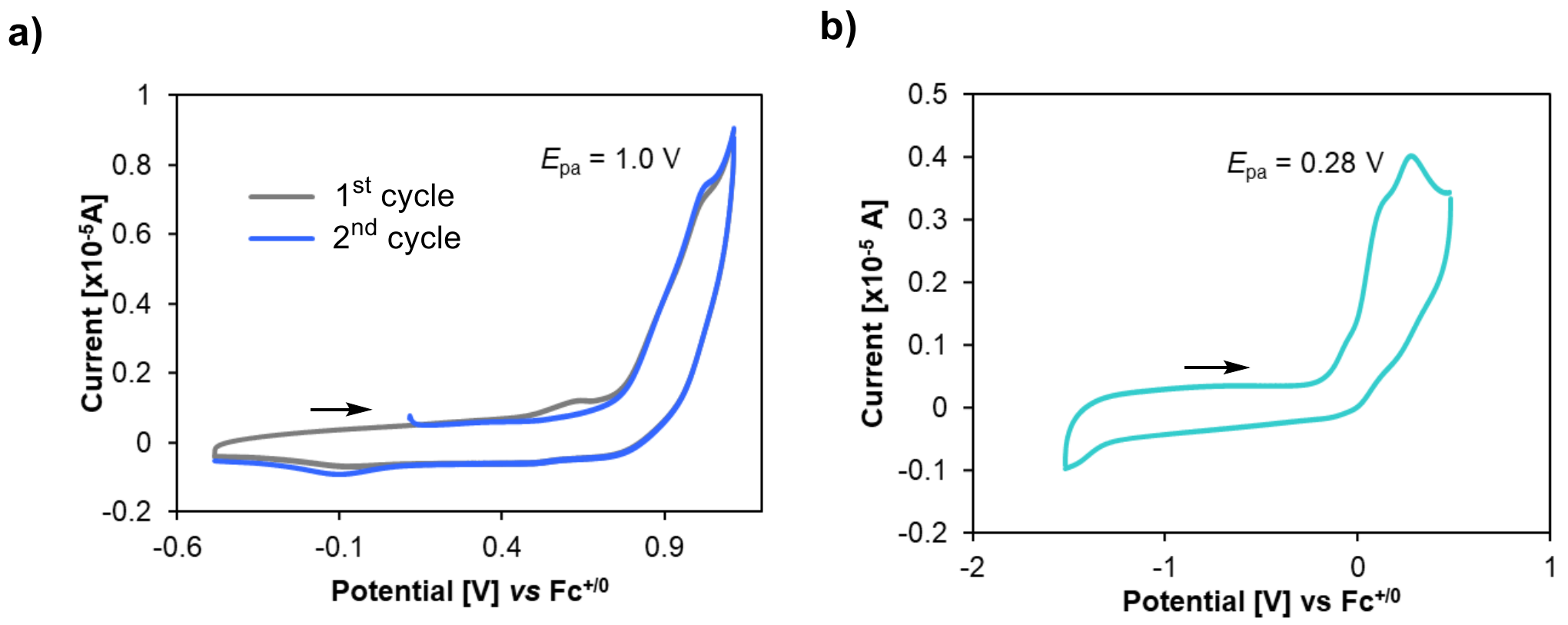

Figure S8. CVs of complexes a) **1** and b) **[1]**$^-$ (generated *in situ* [10^{-3} M] upon deprotonation with KO*t*Bu (6 eq) in DCM and THF, respectively recorded at 100 mV/s. The irreversible nature of the oxidation is retained even at higher potentials (up to 2V).

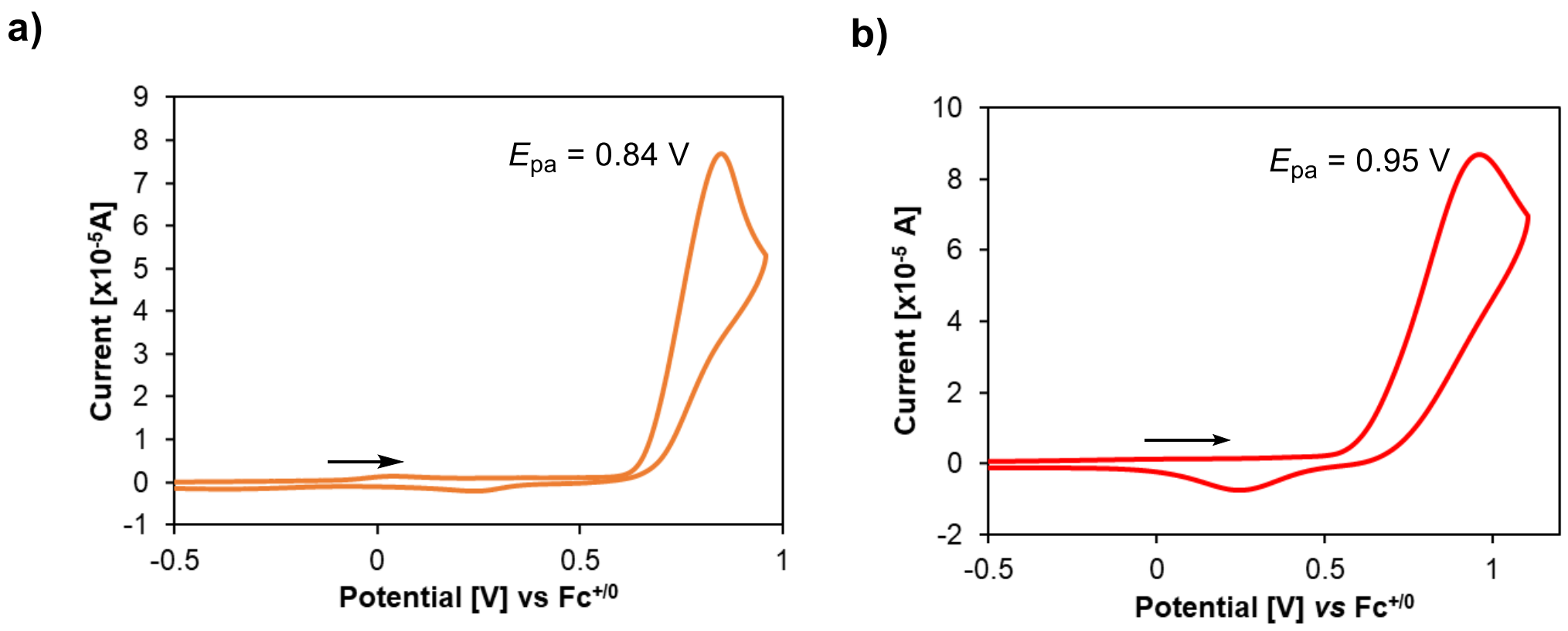

Figure S9. CVs in MeCN ([10^{-2}M], scan rate = 100 mV/s) of a) **3-CA** and b) **3-BZCA**. The irreversible nature of the oxidation is retained even at high scan rates (up to 2 V/s).

Photophysical measurements

Emission spectroscopy was measured on a spectrofluorometer FS5 from Edinburgh instruments® equipped with ozone-free Xenon arc lamp and a single photon counting. Samples of MeCN solutions of 3-benzoylacoumarinamide (**3-BZCA**), 3-coumarinaminde **(3-CA**) and complexes **1** and **[1]**$^-$ were prepared inside a N_2-filled glovebox using anhydrous and degassed solvent unless otherwise is stated. For measurements at room temperature, a 1 cm quartz cuvette was filled with 3 mL of the corresponding solution and tightly closed with a screw-cap lid. The emission spectra were measured outside the glovebox. For measurements at 77 K, outside the glovebox, an NMR tube was filled with 0.5 mL of the corresponding solution and frozen by placing into liquid N_2. The sample was placed inside a quartz tube contained in a liquid N_2 Dewar incorporated into the module sampler (SC-70 Liquid Nitrogen EPR Dewar)**.**

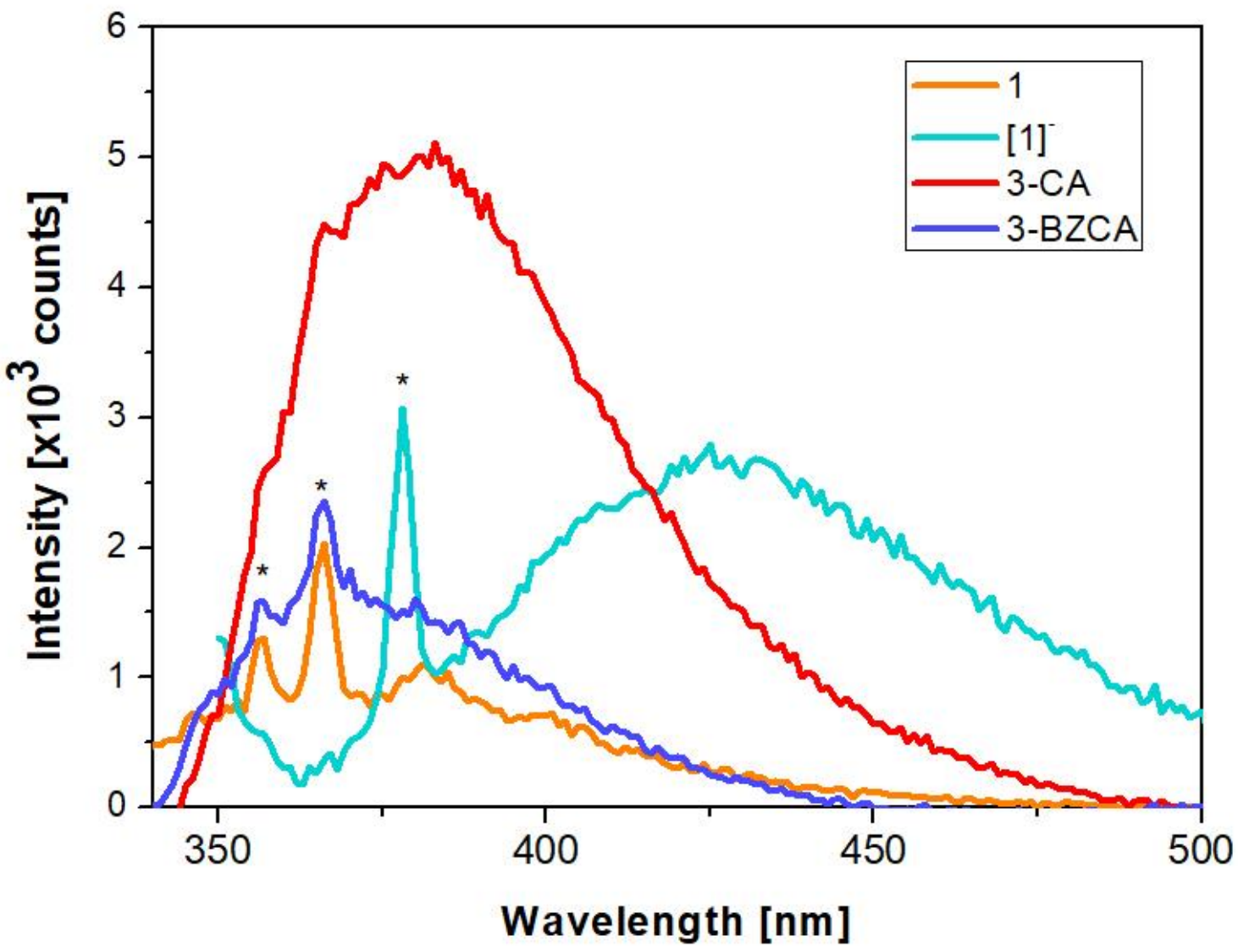

Figure S10. Emission spectra for **1** (7.6 µM, λ_{exc}= 330 nm), **[1]**$^-$ (7.6 µM, λ_{exc}= 340 nm), **3-BZCA** (2 µM, λ_{exc}= 329 nm) and **3-CA** (2 µM, λ_{exc}= 330 nm) in MeCN (1, 3-CA and 3-BZCA) and THF (**[1]**$^-$) at 298K. (*) Raman scattering from the solvent,

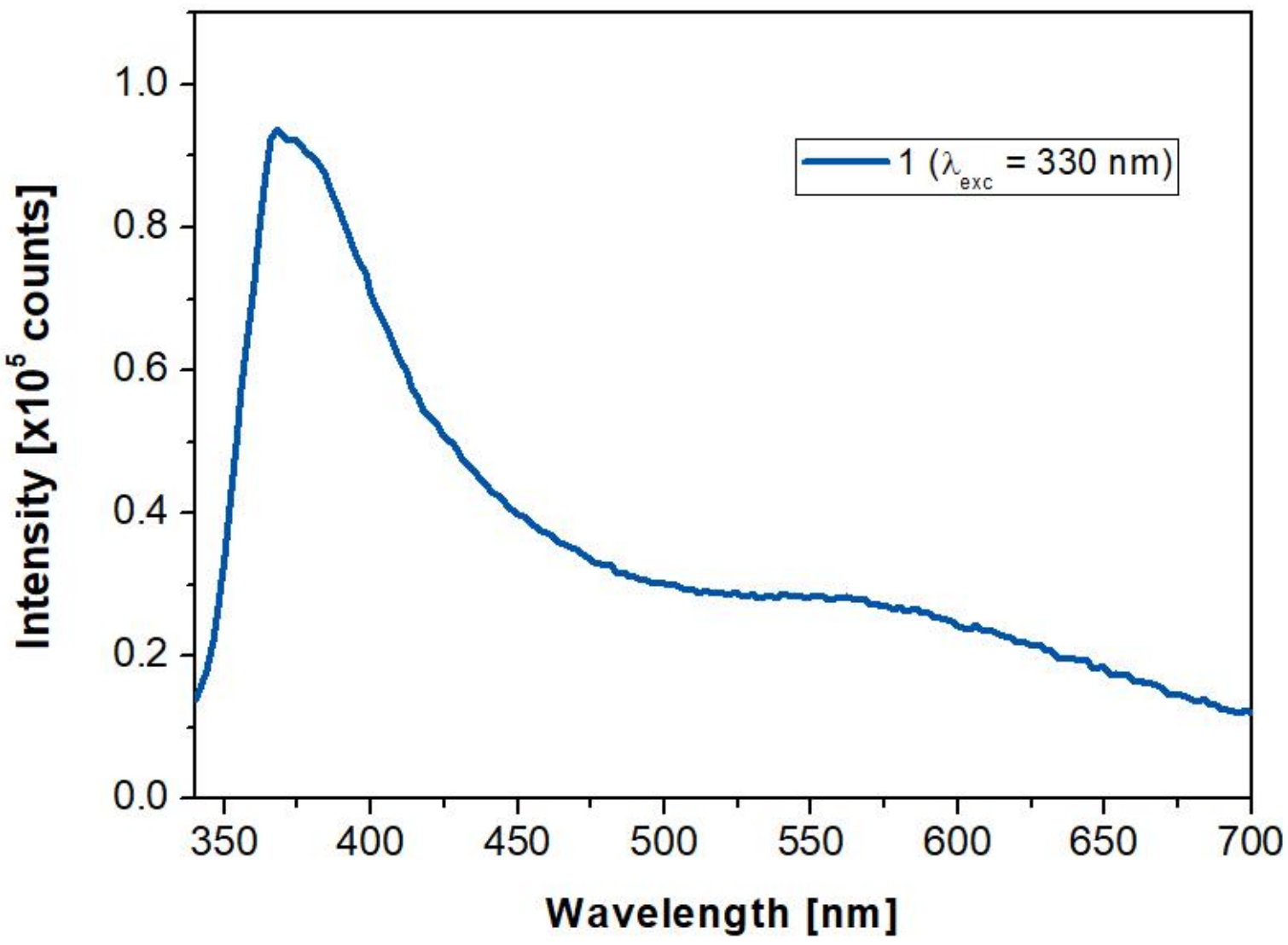

Figure S11. Emission spectrum of **1** (7.6 µM, λ_{exc}= 330 nm) at 298 K in CH_2Cl_2.

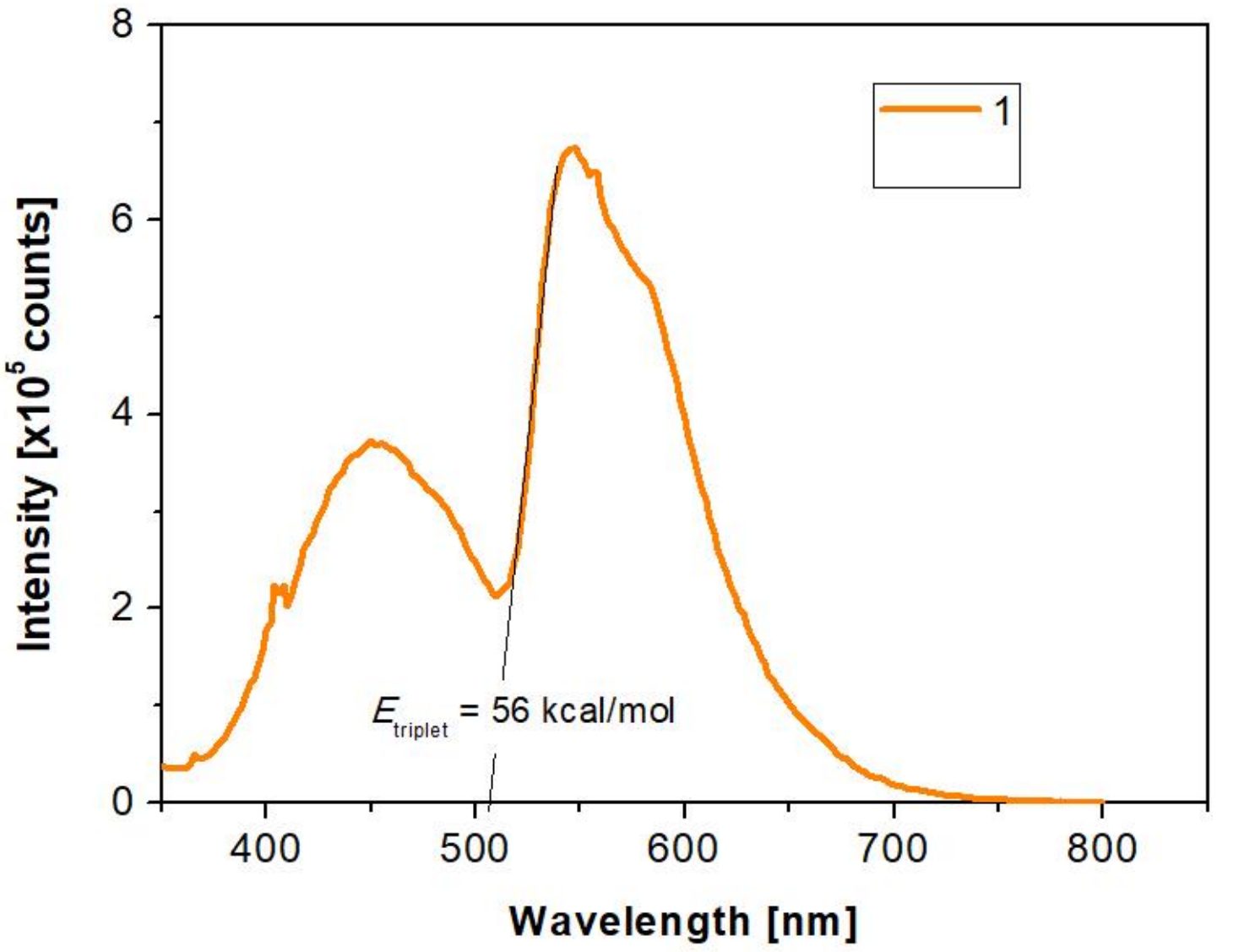

Figure S12. Emission spectrum of **1** (7.6 µM, λ_{exc}= 330 nm) in MeCN at 77 K. The triplet energy E_T was estimated by the intersection of the tangent drawn at the onset of the phosphorescence band.[11]

Redox potentials in the excited state

Redox potentials in the excited state were calculated using the Rehm-Weller approximation, where the $E_{0,0}$ energy was determined from the intersection between the emission and absorption spectra at 298 K. The calculation for E^*_{ox} ($1^{+\bullet}/1^*$) is shown as an example:

$$E^*_{ox}\ (1^{+\bullet}/1^*) = E_{ox}(1^{+\bullet}/1) - E_{0,0}\ (eV)(e) = 0.60\ V - 3.54\ V = -2.84\ V\ vs\ Fc^{+/0}$$

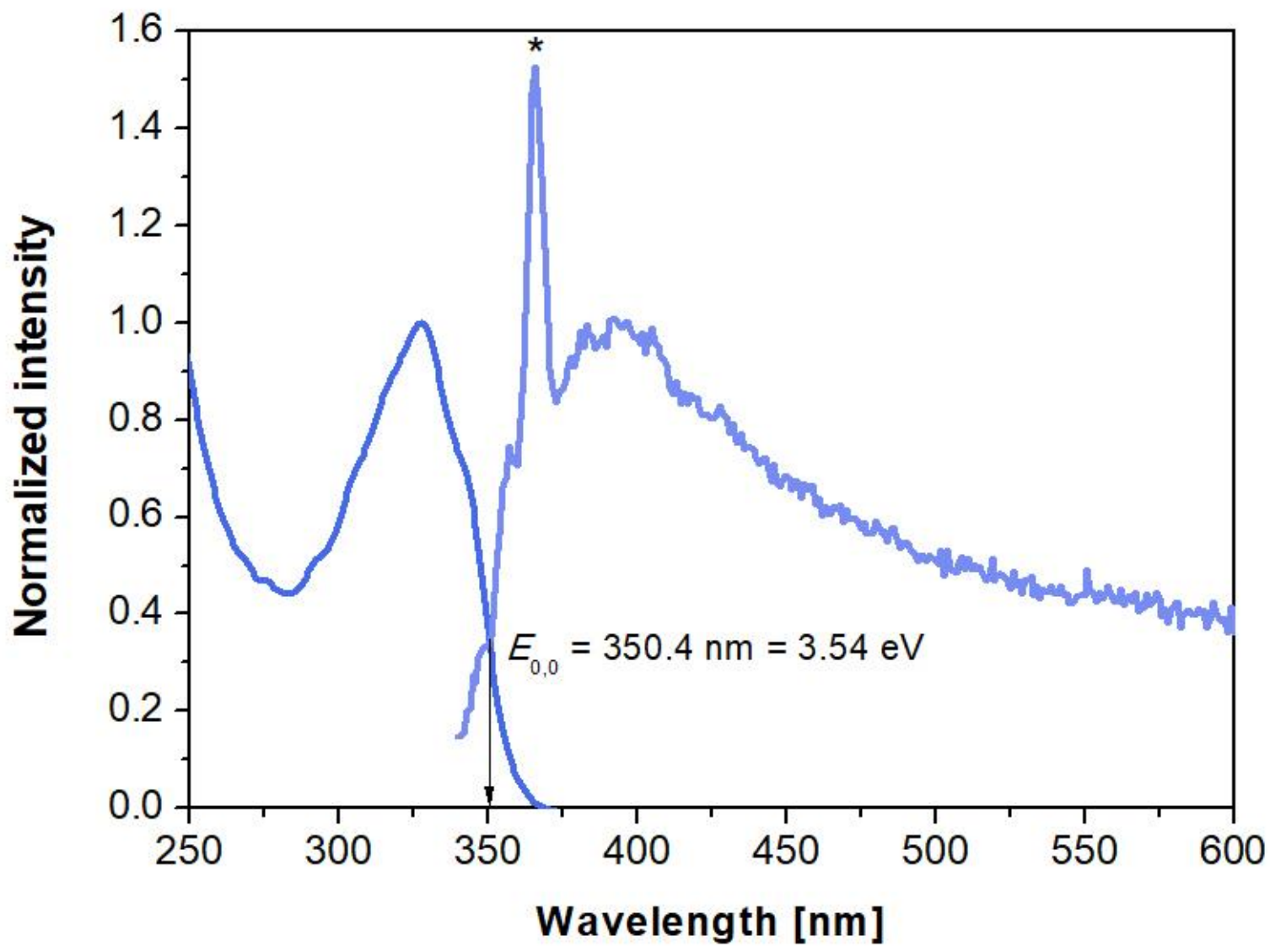

Figure S13. Absorption-emission spectra overlap for **1** in MeCN at 298 K, λ_{exc} = 330 nm. The peak (*) corresponds to Raman scattering from MeCN.

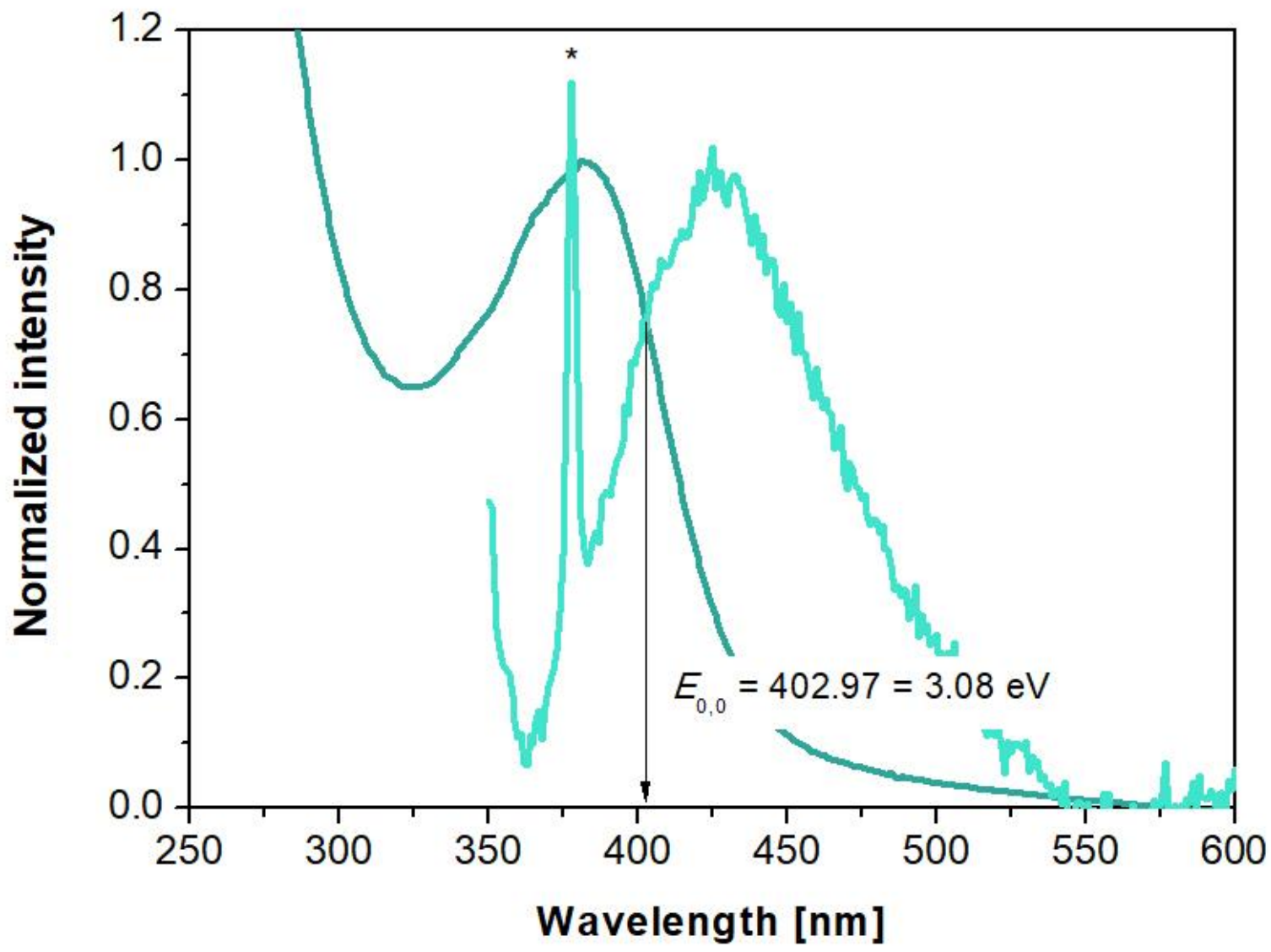

Figure S14. Absorption-emission spectra overlap for **[1]**$^-$, λ_{exc} = 340 nm, generated *in situ* upon addition of 6 equivalents of KOtBu in tetrahydrofuran at 298 K. The peak (*) corresponds to Raman scattering from MeCN.

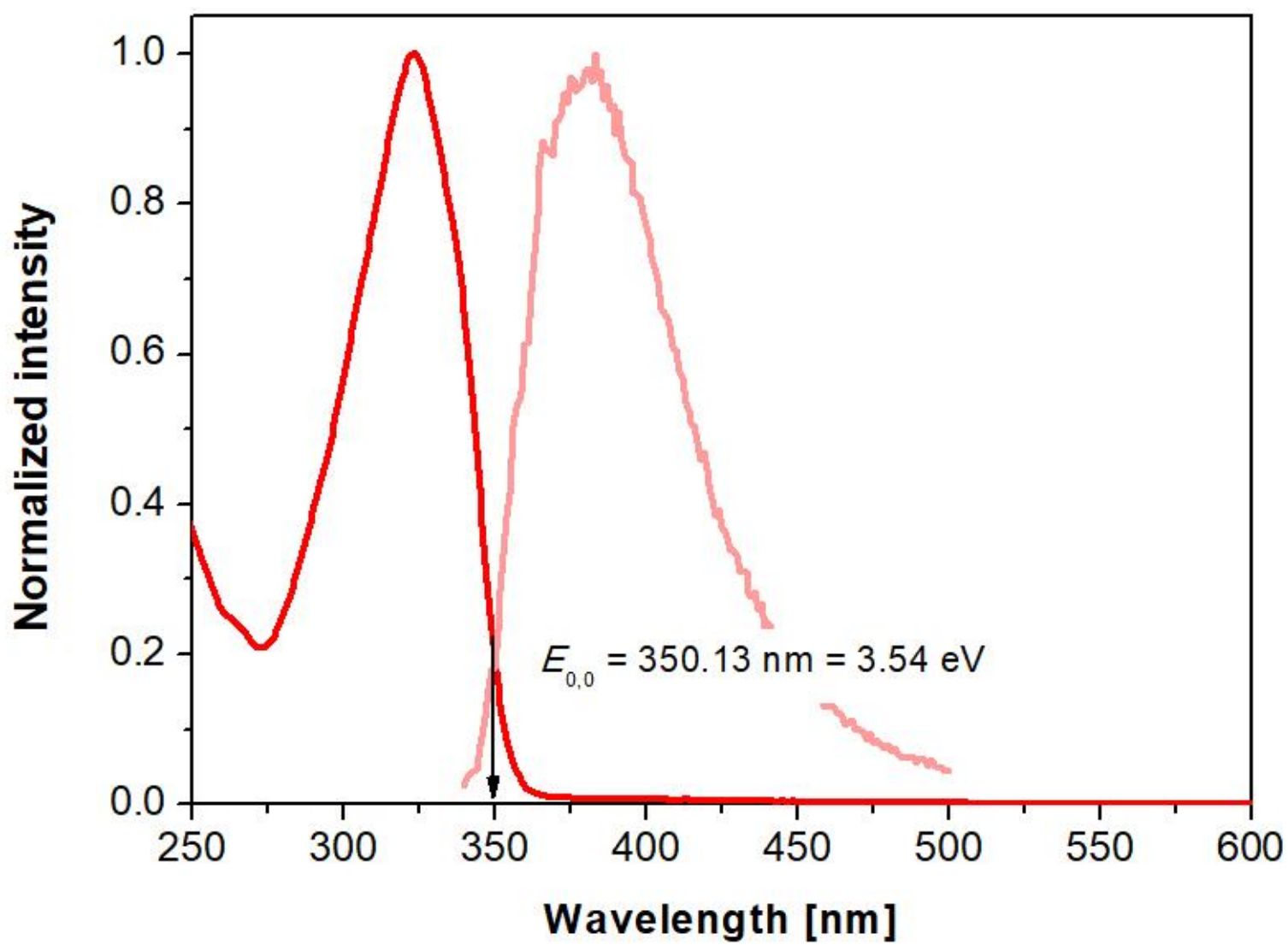

Figure S15. Absorption-emission spectra overlap for **3-CA** in MeCN at 298 K, λ_{exc} = 330 nm.

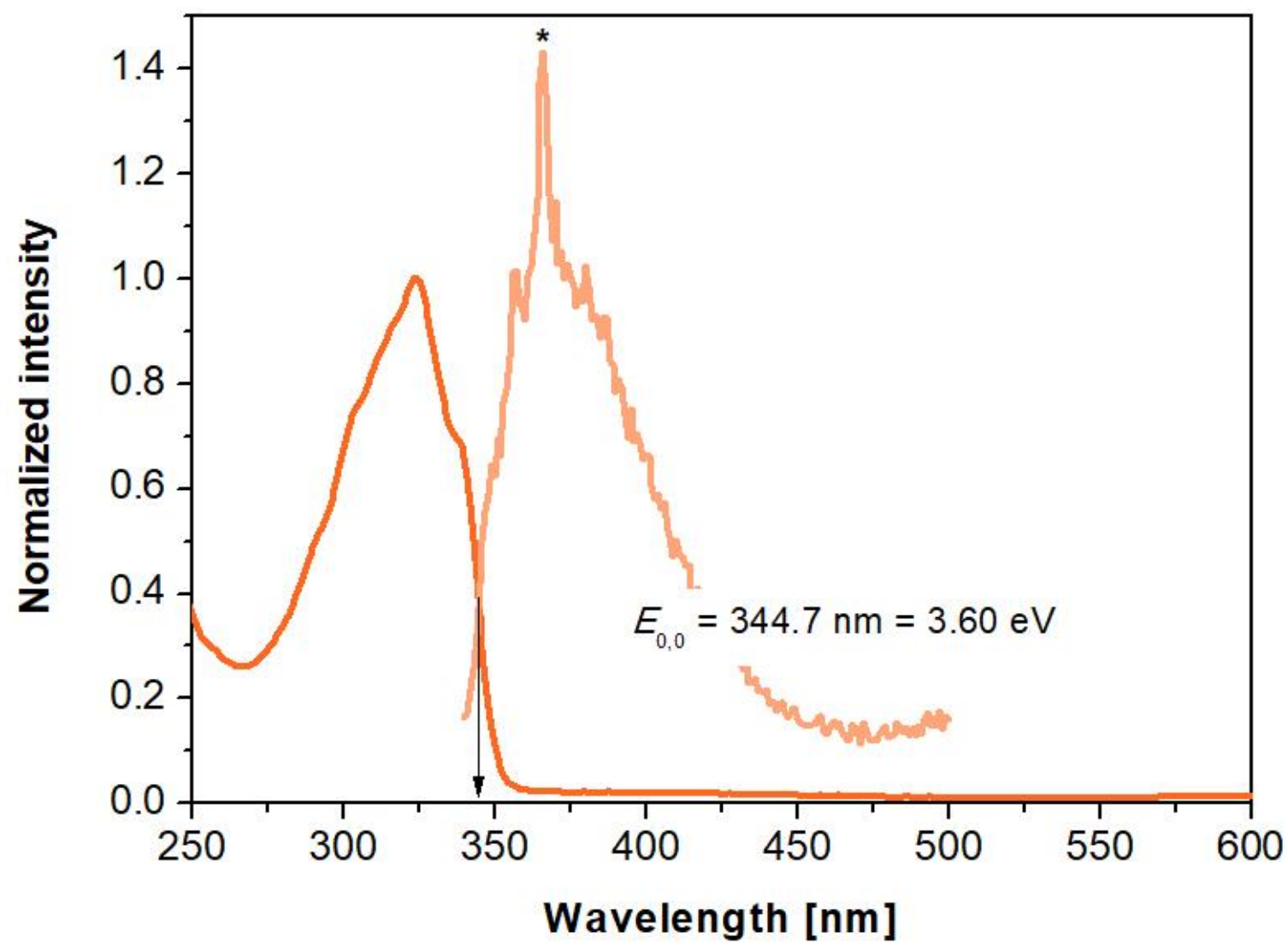

Figure S16. Absorption-emission spectra overlap for **3-BZCA** in MeCN at 298 K, λ_{exc} = 329 nm. The peak (*) corresponds to Raman scattering from MeCN.

Quenching experiments

For quenching experiments, a 1 cm quartz cuvette was filled with 3 mL of a solution of **1** (0.037 mM) in acetonitrile, unless otherwise stated, and closed tightly. Known volumes of a stock solution (10, 20, 30, 40, 50 and 60 µL) of either *p*-nitrophenyl diazonium tetrafluoroborate (A^{NO2}), 2-(4-tolylethynyl)phenol (*B*) or phenyl boronic acid (*C*) (0.011 M in MeCN) were injected into the cuvette under a stream of N_2, and the solution was stirred vigorously. Then, the emission spectra were recorded immediately (λ_{exc} = 330 nm) at 298 K.

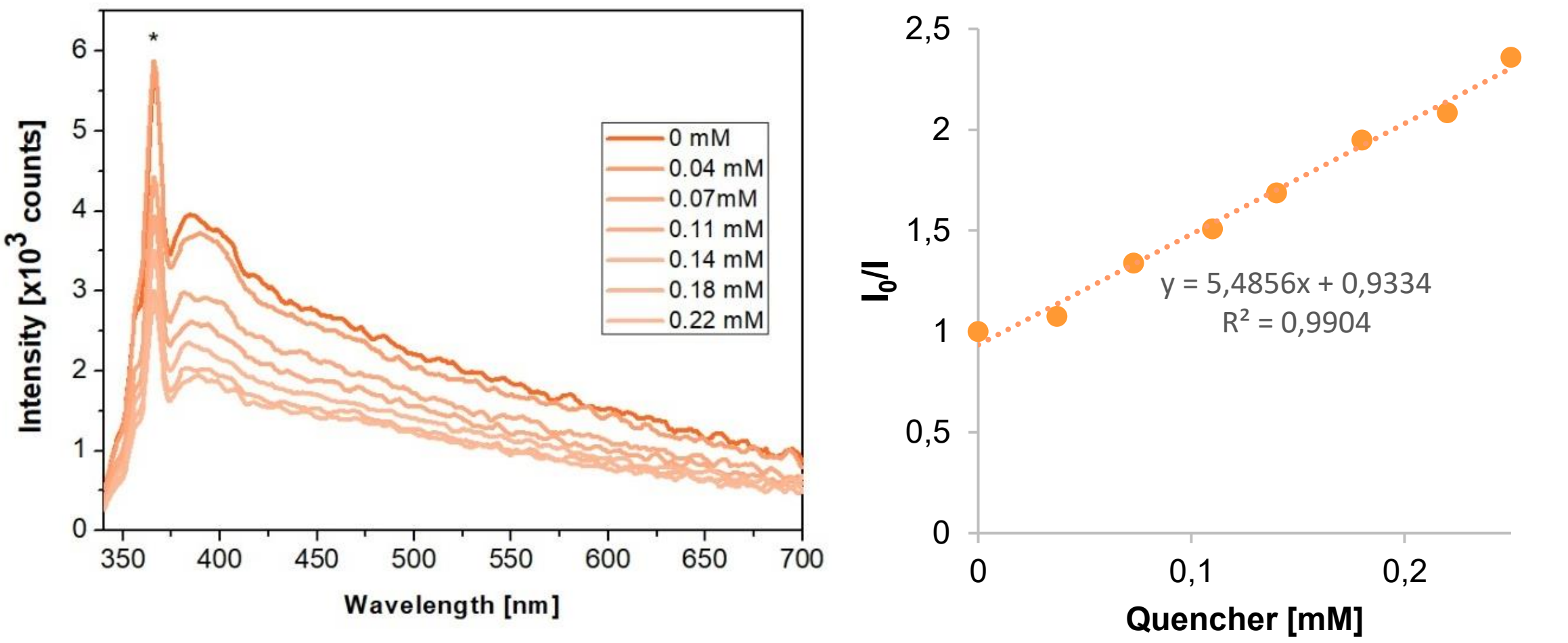

Figure S17.Stern-Volmer quenching experiments of **1** in the presence of *p*-nitrophenyl diazonium tetrafluoroborate (A^{NO2}) in MeCN. A linear response is observed upon addition of the quencher. After linear fitting, a K_{SV} = 5.49 mM^{-1} was obtained from the slope. The peak (*) corresponds to Raman scattering from MeCN.

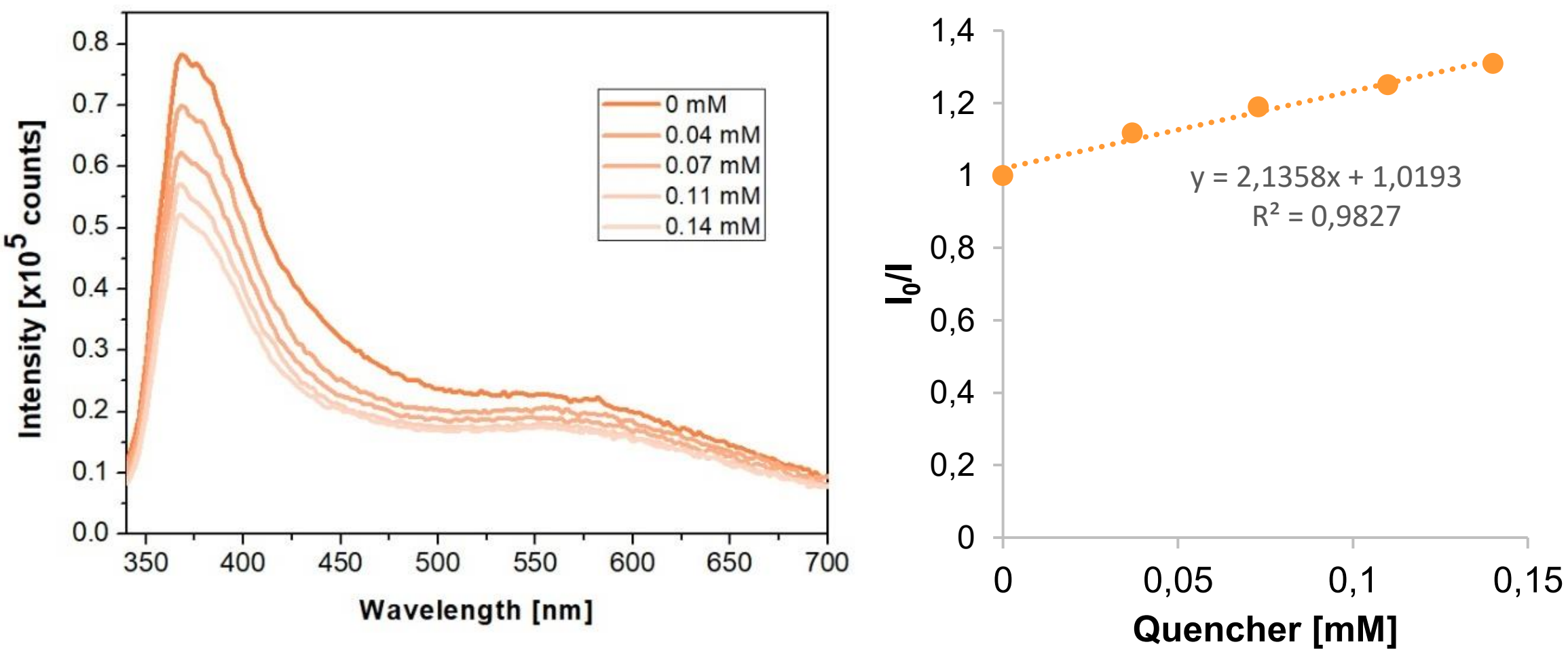

Figure S18. Stern-Volmer quenching experiments of **1** in the presence of *p*-nitrophenyl diazonium tetrafluoroborate (A^{NO2}) in CH_2Cl_2. A linear response is observed upon addition of the quencher. After linear fitting, a K_{SV} = 2.14 mM^{-1} was obtained from the slope.

a)

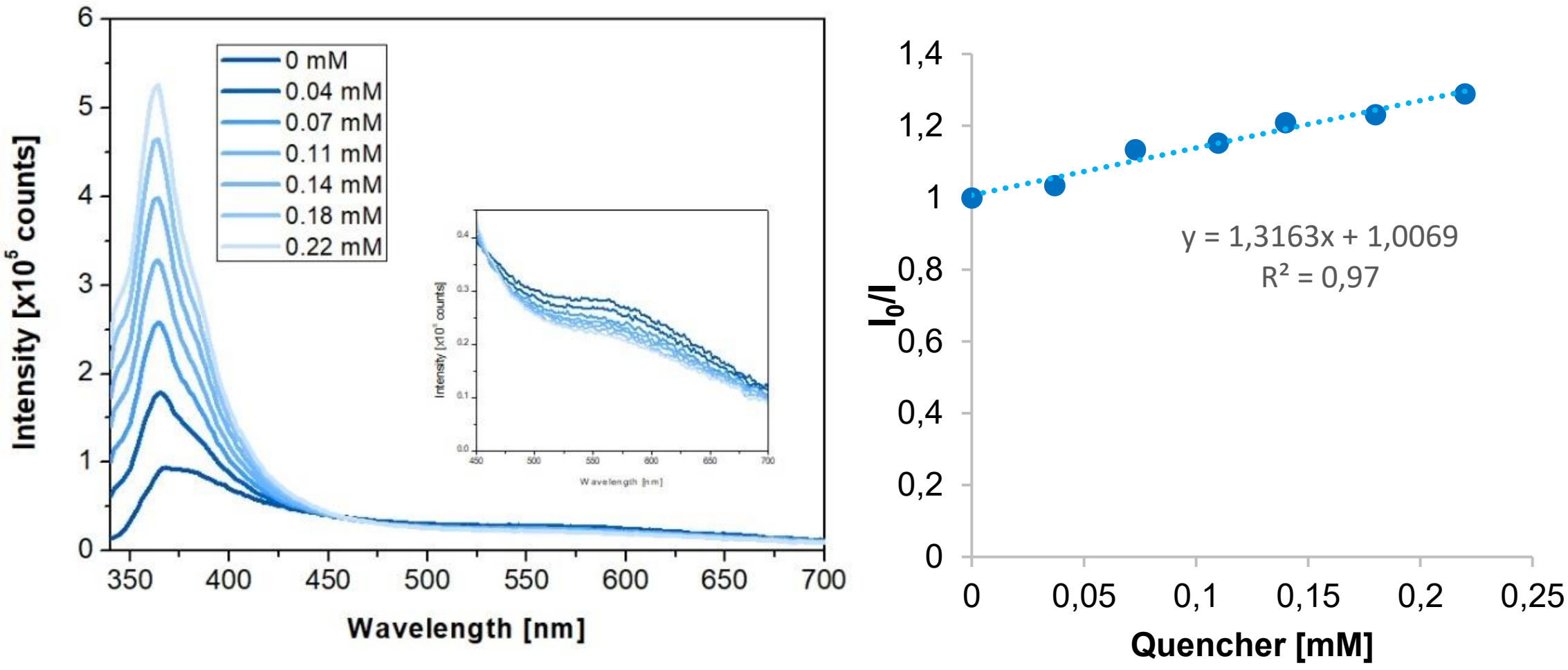

b)

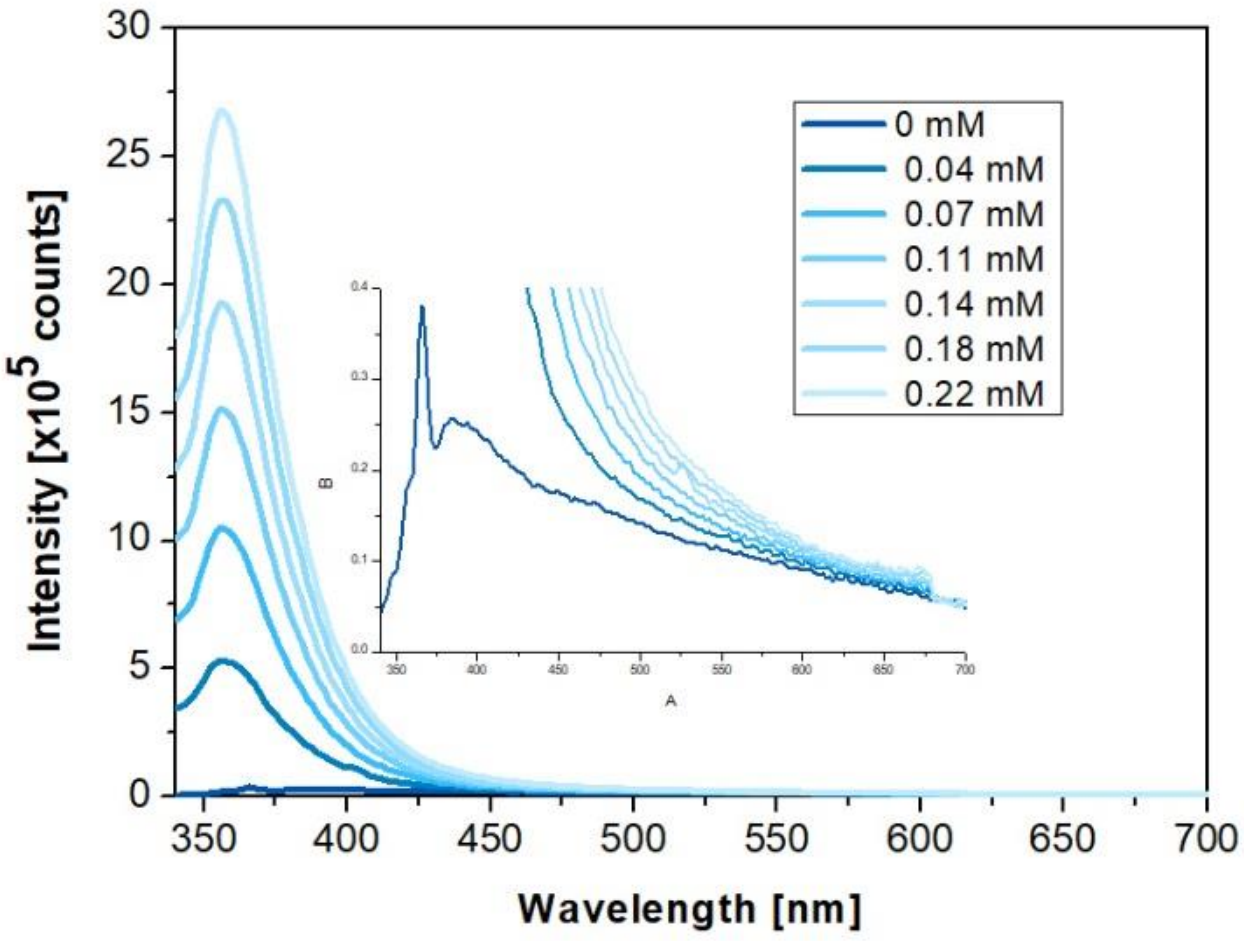

Figure S19. a) Stern-Volmer quenching experiments of **1** in the presence of 2-(4-Tolylethynyl)phenol (*B*) in CH_2Cl_2. Given substrate's emission at 360 nm, changes in emission were monitored at 570 nm. A linear response is observed upon addition of the quencher. After linear fitting, a K_{SV} = 1.32 mM^{-1} was obtained from the slope. b) In MeCN, the fluorescence emission derived from *B* overlaps with the emission from **1**, thus masking the quenching of **1** by B.

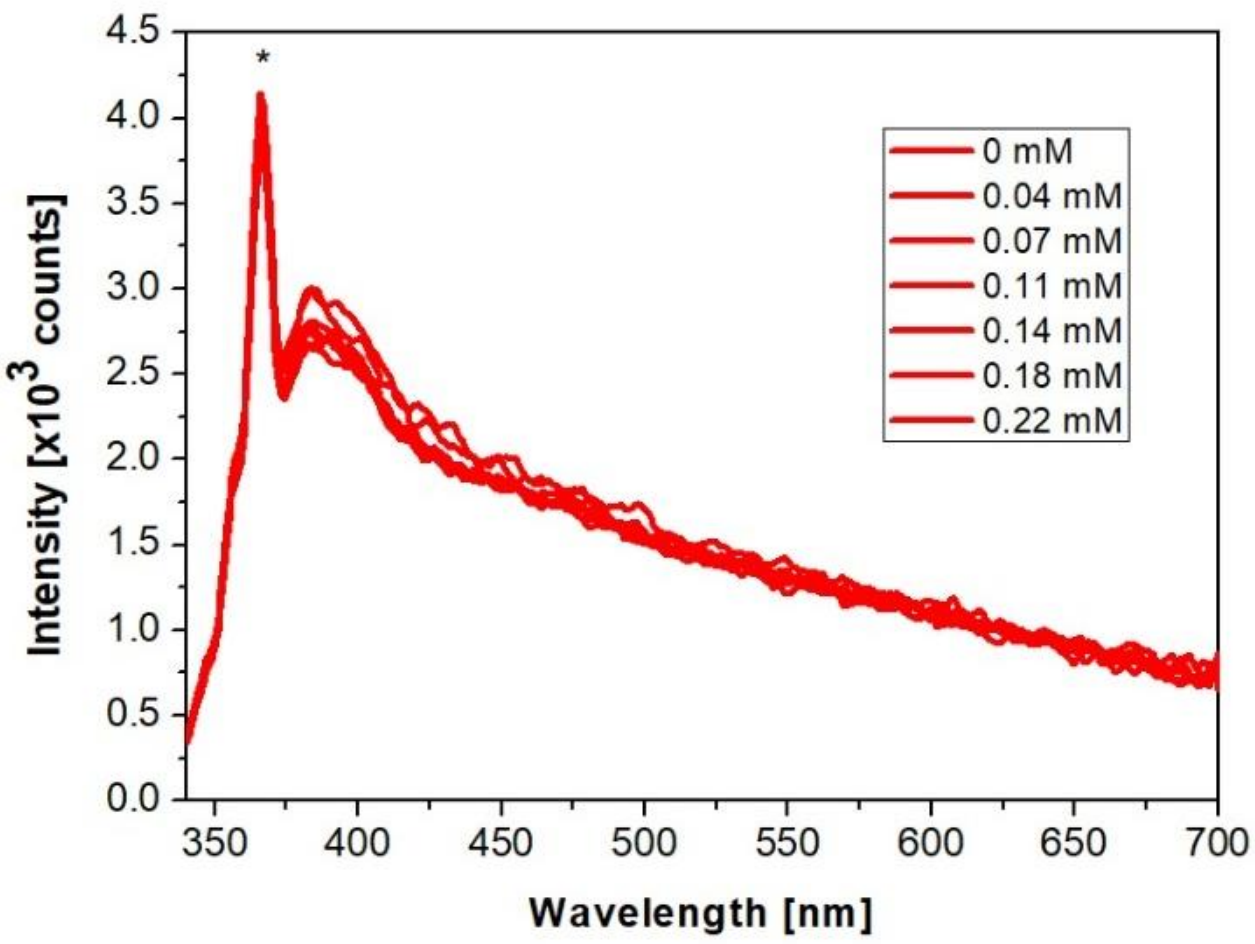

Figure S20. Stern-Volmer quenching experiments of **1** in the presence of phenyl boronic acid (*D*). No drastic changes were observed in fluorescence intensity upon addition of the quencher. The peak (*) corresponds to Raman scattering from MeCN.

Emission lifetime measurements

Phosphorescence decay lifetimes were recorded on a spectrofluorometer FS5 from Edinburgh instruments® equipped with microsecond Xe flashlamp. 0.5 mL of a solution of complex 1 in MeCN (7.6 µM) were added into a NMR-tube. The NMR tube was placed inside a Dewar containing liquid N_2 until the solution was frozen. The sample was placed inside a quartz tube contained in a liquid N_2 Dewar incorporated into the module sampler (SC-70 Liquid Nitrogen EPR Dewar). MCS (multi-channel gated) recording of the phosphorescence decay of **1** was carried out on a spectrofluorometer FS5 from Edinburgh instruments® in frozen (77 K) MeCN. Exponential fitting (Time scan) was carried out using the Fluoracle® Software from Edinburgh instruments®.

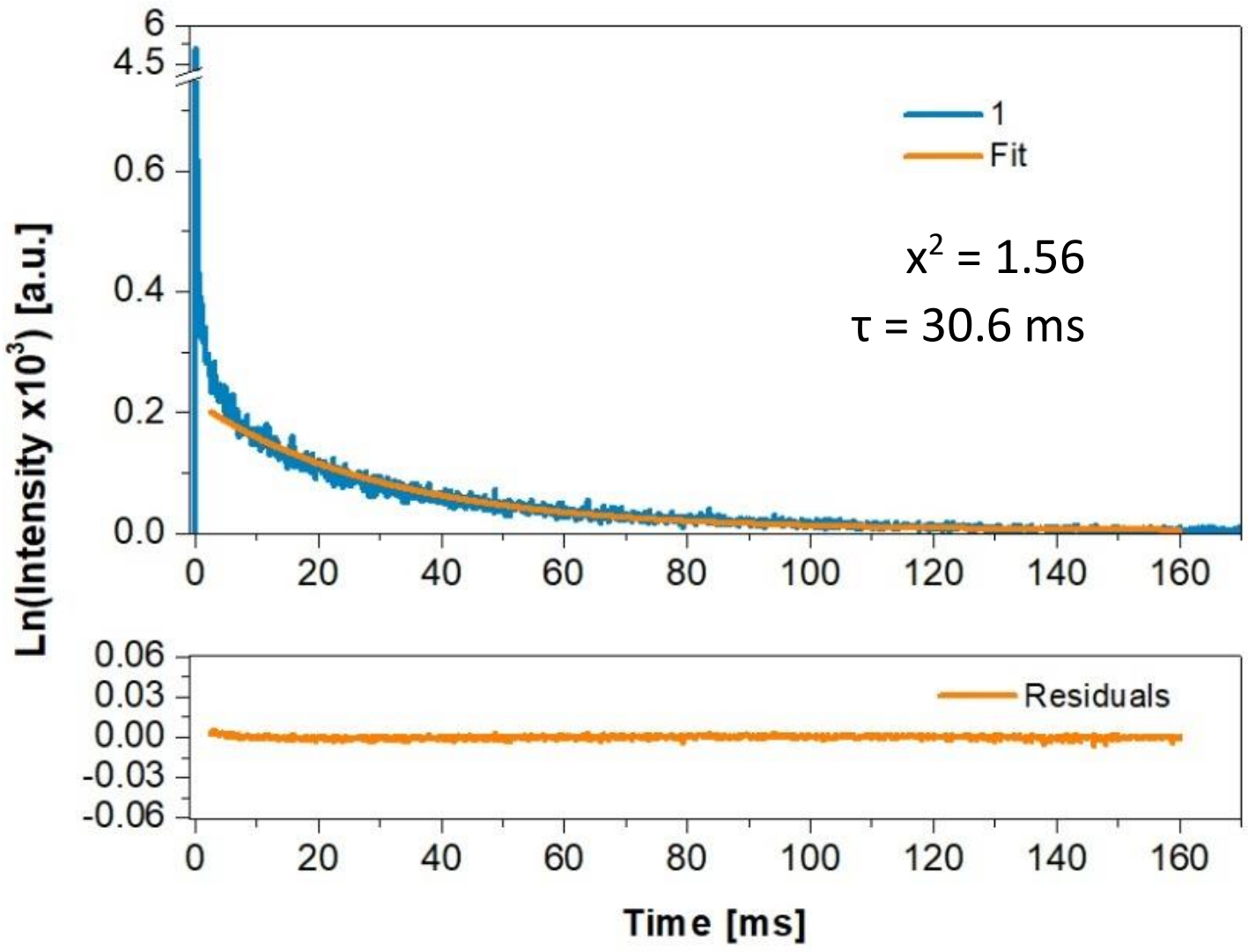

Figure S21. Phosphorescence decay of **1** recorded in MeCN at 77 K and exponential fit (top) and residuals (bottom).

Photocatalytic exploration

Irradiation experiments were conducted using a custom-made irradiation setup, similar to that reported by the König group,[12] with a six-blue LED array connected in series (450 nm, 115 mW of optical power), which allows for the simultaneous irradiation of up to six reactions. This array is embedded in a metallic block that is connected to a water circulation system, allowing the temperature to be kept constant (Figure S16).

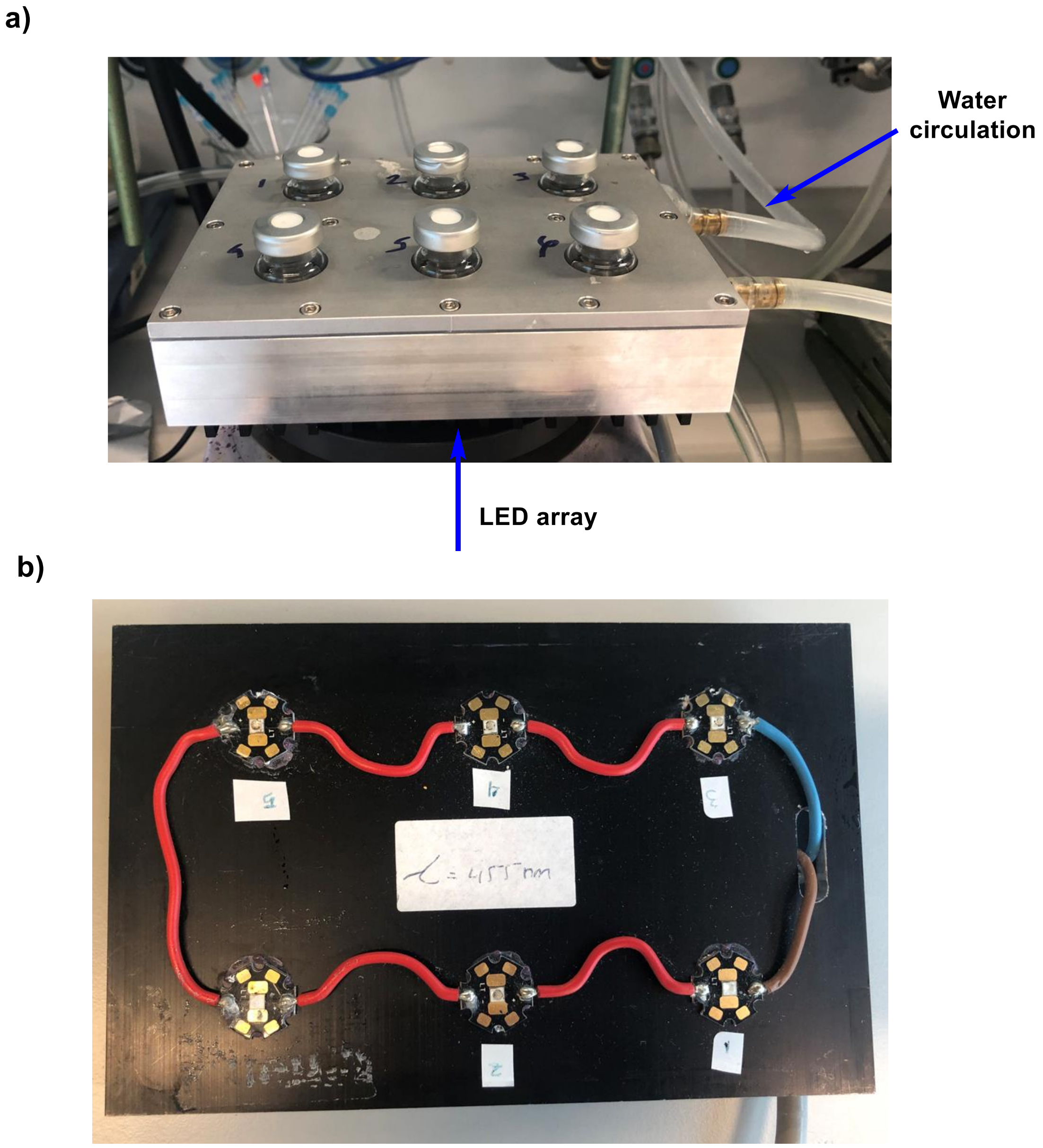

Figure S22. a) Custom-made Irradiation setup. b) LED array.

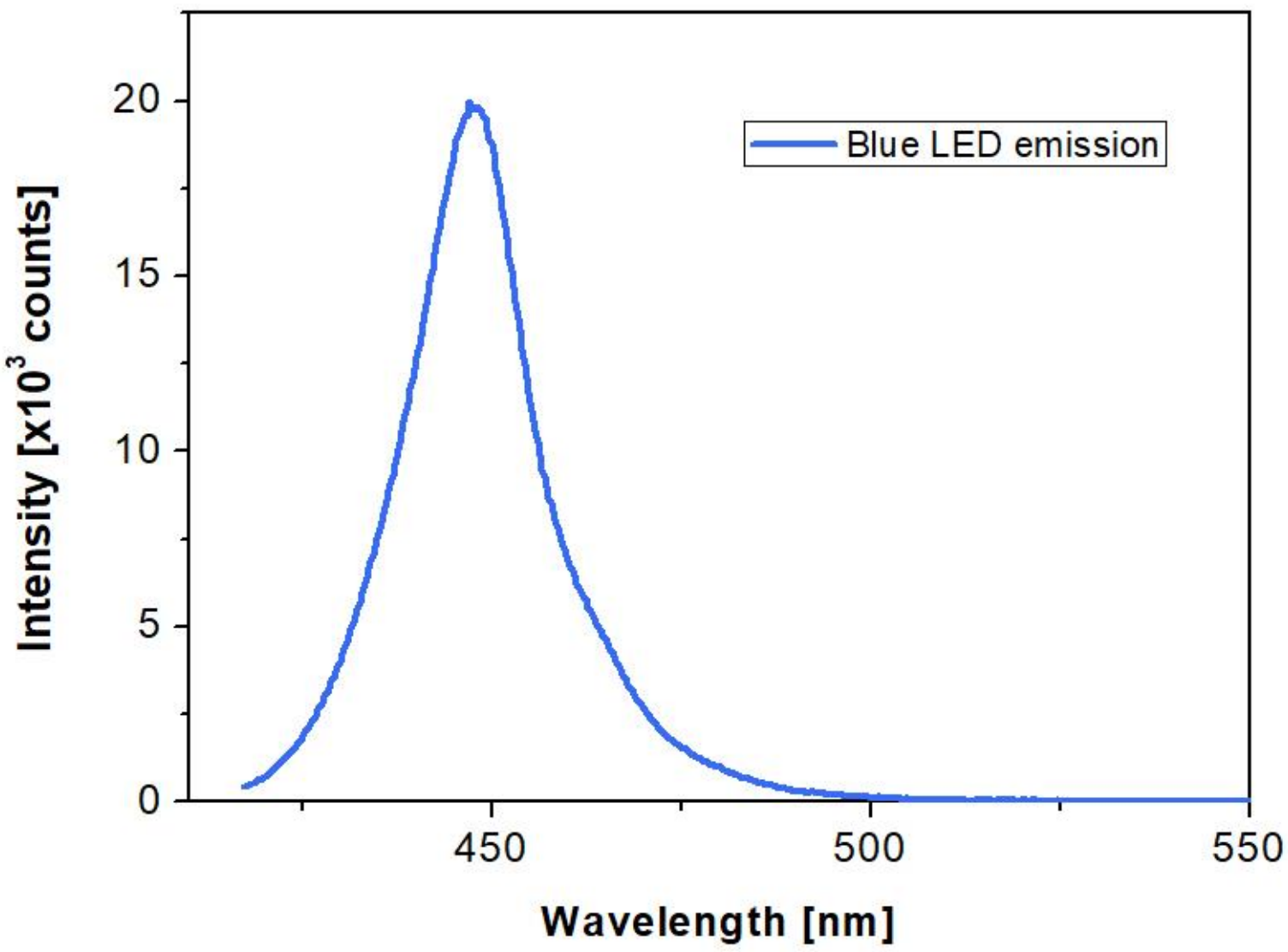

Figure S23. Average emission of the blue light-emitting diodes (λ_{max} = 450 nm) used for photocatalytic experiments.

Diarylation reactions with phenyl boronic acid

The experimental conditions described herein are based on those previously reported by the Fouquet group.[13]

In a 6 mL crimp-top vial equipped with a stirring bar, phenyl boronic (62.5 µmol, 1 eq.), the corresponding diazonium salt (93.75 µmol, 1.5 eq.), CsF (65.63 µmol, 1.05 eq), and the catalyst(s) were added. The vial was sealed and purged with a stream of N_2 for 20 min, then 0.5 mL of anhydrous and degassed MeCN were added. Subsequently, the mixture was stirred at room temperature for 16 h while irradiating with blue LEDs (455 nm). After this time, the reaction was stopped and the solution passed through a celite pad (0.5 cm) and rinsed with acetonitrile (3 x 1 mL). Upon evaporation, 3,5-dinitro-methylbenzoate was added as internal standard, and the mixture was redissolved in 0.5 mL of DMSO-d_6. Figure S18, shows a representative example of the identification and quantification of the products derived from the catalytic trials.

4-nitro-1,1'-biphenyl. ^{1}H NMR (DMSO-d_6, 25 °C, 600MHz) δ/ppm: 7.46 (t, 1 H, *p*-CH Ph), 7.52 (t, 2 H, *m*-CH Ph), 7.76 (d 2 H, *o*-CH Ph), 7.93 (d, 2 H, *o*-CH *p*-NO_2Ph), 8.28 (d, 2 H, *m*-CH *p*-NO_2Ph). Similar to the previously reported.[13]

4-methoxy-1,1'-biphenyl. ^{1}H NMR (DMSO-d_6, 25 °C, 600MHz) δ/ppm: 3.78 (s, 1H, CH_3)7.00 (d, 2 H, *o*-CH *p*-MeO-Ph), 7.33 (*m*-CH Ph), 7.41(t, 1H, *p*-CH Ph)7.58 (d 2 H, *m*-CH *p*-MeO-Ph), 7.76(d, 2 H, *o*-CH Ph). Similar to the previously reported.[13]

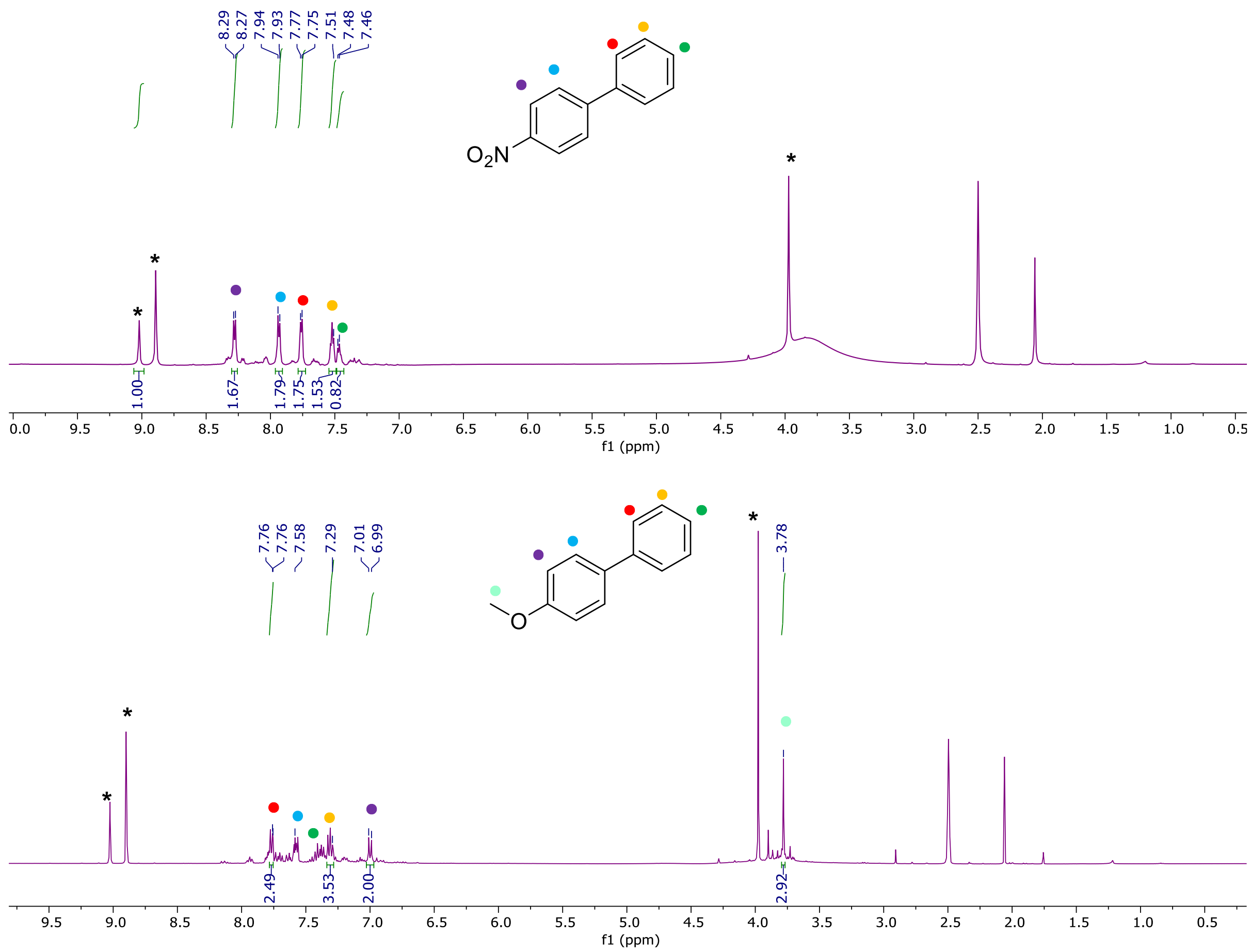

Figure S24. ^{1}H NMR of the reaction between phenyl boronic acid and *p*-nitrophenyl diazonium tetrafluoroborate (top) or p-methoxy phenyl diazonium tetrafluoroborate (bottom) when complex **1** is used as a catalyst (10%) in DMSO-d$_6$. (*) signals of the internal standard.

Arylative cyclization of 2-(4-tolylethynyl)phenol

The experimental conditions described herein are based on those previously reported.[4]

In a 6 mL crimp-top vial equipped with a stirring bar, 2-(4-tolylethynyl)phenol (50 µmol, 1 eq.), the corresponding diazonium salt (100 µmol, 2 eq.), $NaHCO_3$ (200 µmol, 4 eq), and the catalyst(s) were added. The vial was sealed and purged with a stream of N_2 for 20 min. Then, 1 mL of dry and degassed MeCN was added. The mixture was stirred at room temperature for 16 h while irradiating with blue LEDs (455 nm). Subsequently, the reaction was passed through a celite pad (1 cm) and rinsed with acetonitrile (3 x 1 mL). Upon evaporation, 3,5-dinitro-methylbenzoate was added as internal standard, and the mixture was redissolved in 0.5 mL of DMSO-*d*$_6$. Figure S19, shows a representative example of the identification and quantification of the products derived from the catalytic trials.

3-(4-Nitrophenyl)-2-(*p*-tolyl)benzofuran. ^{1}H NMR (DMSO-*d*$_6$, 25 °C, 600MHz) δ/ppm: 2.32 (s, 3 H, CH_3, Tol), 7.22 (d, 2 H, *o*-CH Tol), 7.31 (t, 1 H, 5-CH benzofuran), 7.41 (t, 1 H, 6-CH benzofuran), 7.44 (d 2 H, *o*-CH Ph), 7.51 (d, 1 H, 4-CH benzofuran), 7.65-7.69 (m, 3 H, *o*-CH *p*-NO_2Ph, 7-CH benzofuran), 8.33 (d 2 H, *m*-CH *p*-NO_2Ph). Spectroscopic data matched the previously reported data.[4]

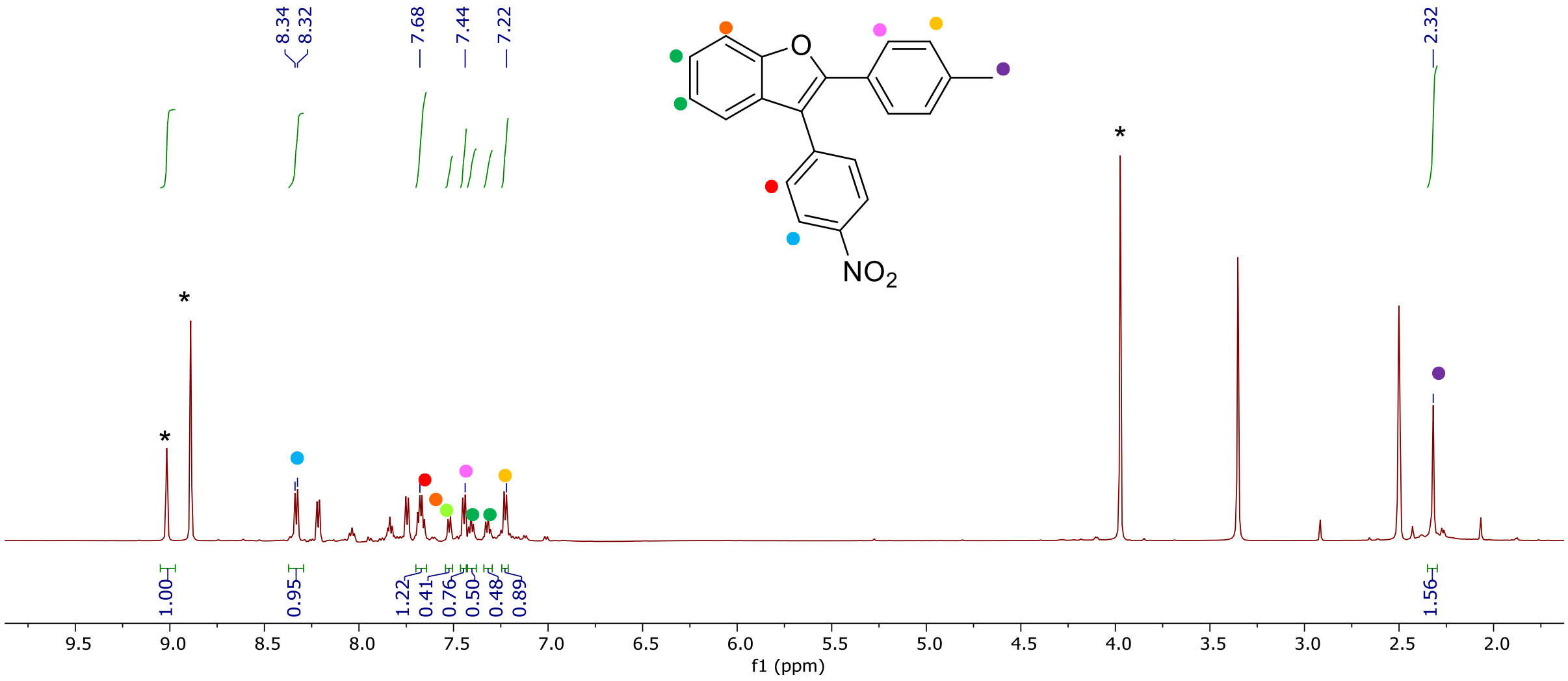

Figure S25. ^{1}H NMR of the reaction between 2-(4-tolylethynyl)phenol and *p*-nitrophenyl diazonium tetrafluoroborate when complex **1** is used as catalyst (5%) in DMSO-d$_6$. (*) signals of the internal standard.

Control experiments

__Diarylations__. The reactions were conducted in duplicate following the procedure previously described.

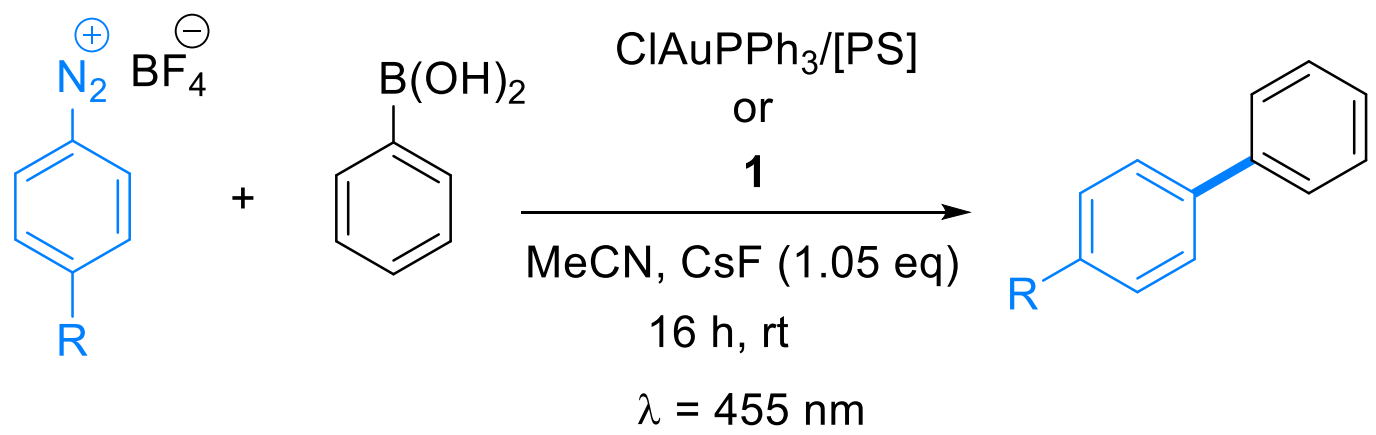

Table S2. ^{1}H NMR yields for the diarylation reaction carried out with *p*-nitrophenyl diazonium tetrafluoroborate.

**Entry**	**Conditions**	**Yield (%)**								
		**Cross-coupling**			**Nitrobenzene**			**Biphenyl**		
		**1**	**2**	**Average**	**1**	**2**	**Average**	**1**	**2**	**Average**
1	No catalysts	18	14	16	24	29	37	2	2	2
2	1 (10%) Dark	34*		34	1*		1	12*		12
3	1 (10%) Dark 28ºC	13		13	10		10	2		2
4	1 (10%)	62	63	63	2	6	4	2	2	2
5	1 (10%)[b]	37		37	2			2		
6	Cl-Au-PPh$_3$ (10%) 3-CA (10%)	37	49	43	12	15	14	8	13	10
7	Cl-Au-PPh$_3$ (10%) 3-BZCA (10%)	18	20	19	15	21	18	10	11	11
8	Cl-Au-PPh$_3$ (10%)	36*	32	34	6*	9	8	6*	5	6
9	Cl-Au-PPh$_3$ (10%), [Ru(bpy)$_3$][PF$_6$]$_2$ (2%)	38	38	38 (75)[a]	9	4	6	10	4	7
10	Cl-Au-PPh$_3$ (10%), Acridinium (4%)	36	33	35 (76)[a]	18	16	17	9	8	8
11	Cl-Au-PPh$_3$ (10%), 3-CA (4%)	40	46	43	20	11	15	8	12	10
12	Cl-Au-PPh$_3$ (10%), 3-CA (4%) Dark	20	37	28	1	4	2	9	16	13
13	3-CA(10%)	7	8	8	32	12	22	<1	<1	<1
14	3-BZCA (10%)	7	8	7	19	5	12	<1	<1	<1

[a]Reported isolated yield.[13]

[b] Carried out at 365 nm.

*TCCE used as internal standard.

Table S3. ^{1}H NMR yields for the diarylation reaction carried out with *p*-methoxyphenyl diazonium tetrafluoroborate.

**Entry**	**Conditions**	**Yield (%)**								
		**Cross-coupling**			**Anisole**			**Biphenyl**		
		**1**	**2**	**Average**	**1**	**2**	**Average**	**1**	**2**	**Average**
1	No catalysts	5	2	3	2	1	1	<1	<1	<1
2	1 (10%) Dark	4	5	4	1	1	11	5	6	5
3	1 (10%)	35	34	35	8	2	5	13	11	12
4	Cl-Au-PPh_3 (10%) 3-CA (10%)	31	38	34	1	3	2	5	8	7
5	Cl-Au-PPh_3 (10%) 3-BZCA (10%)	28	40	34	2	6	4	9	11	10
6	Cl-Au-PPh_3 (10%)	34	46	40	1	2	1	8	7	7
7	Cl-Au-PPh_3 (10%), 3-CA (4%)	43	51	47	3	2	3	7	6	6
8	Cl-Au-PPh_3 (10%), 3-CA (4%) Dark	5	5	5	<1	<1	<1	5	6	5
9	Cl-Au-PPh_3 (10%), [Ru(bpy)$_3$][PF_6]$_2$ (2%)	42	54	48 (63)[a]	4	4	4	4	13	9
10	Cl-Au-PPh_3 (10%), Acridinium (4%)	47	51	49 (55)[a]	4	1	3	8	9	8
11	3-CA (10%)	<1	3	2	<1	<1	<1	<1	<1	<1
12	3-BZCA (10%)	<1	<1	<1	<1	<1	<1	<1	<1	<1

[a]Reported isolated yield.[13]

Arylative cyclizations

The reactions were conducted according to the previously described procedure.

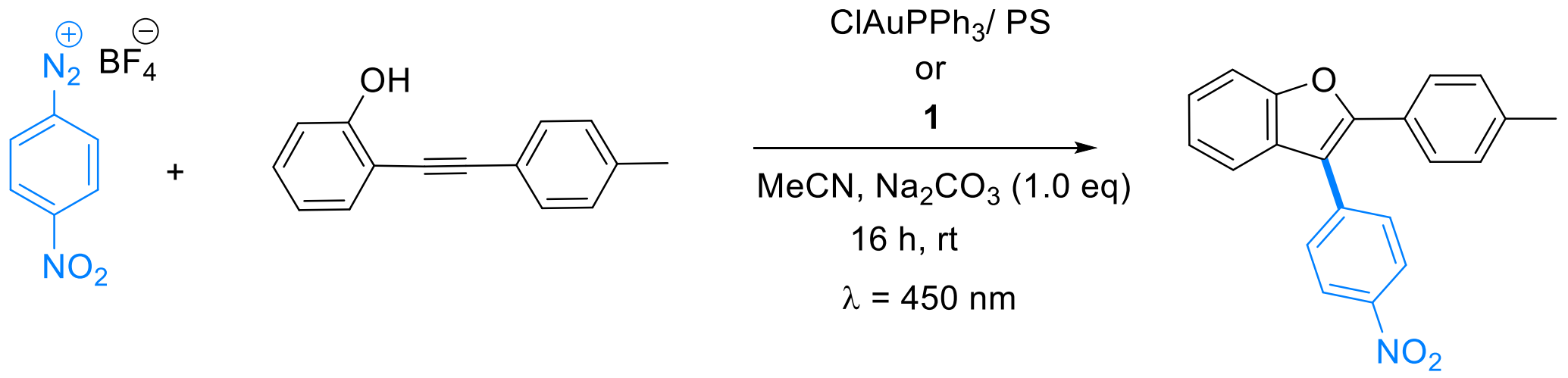

Table S4. ^{1}H NMR yields for the arylative cyclization reaction carried out with *p*-nitro diazonium tetrafluoroborate and 2-(4-tolylethynyl)phenol.

**Entry**	**Conditions**	**Yield (%)**								
		**Cross-coupling**			**Nitrobenzene**			**Protodeauration**		
		**1**	**2**	**Average**	**1**	**2**	**Average**	**1**	**2**	**Average**
1	No catalysts	8	10	9	16	18	17	1	2	1
2	1 (5%)	48	53	51	33	16	25	<1	<1	<1
3	1 (5%)[a]	48			50			<1		
4	1 (5%) Dark	12	8	10	16	11	14	24	<1	
5	1 (5%) Dark 28ºC	17		17	4		4	<1		<1
6	Cl-Au-PPh_3 (5%), 3-CA (5%)	40	31	36	18	21	19	<1	<1	<1
7	Cl-Au-PPh_3 (5%), 3-BZCA (5%)	4			10			7		
8	Cl-Au-PPh_3 (5%)	43	40	42(32)[b]	19	20	19	<1	<1	<1
9	3-CA (5%)	8	14	11	19	18	19	<1	<1	<1
10	3-BZCA (5%)	8			28			2		

[a] Experiment recorded under irradiation at 365 nm. [b]Reported NMR yield from ref [4].

Temperature monitoring

We monitored temperature changes on the reactions catalyzed by **1** over 16 h to exclude any potential (photo)thermal activation. The samples were prepared in a 1 cm cuvette under an N_2 atmosphere, under conditions similar to those used for the photocatalytic trials. For both arylative cyclization (a) and diarylation (b), a subtle increase in temperature was detected (T ≈ 28 °C). The same reactions were studied at a constant temperature (T = 28 °C) in the absence of irradiation to evaluate the influence of temperature on the formation of the cross-coupling product. Quantification by ^{1}H NMR indicated that, in both cases, irradiation is required to afford the desired product in higher yield.

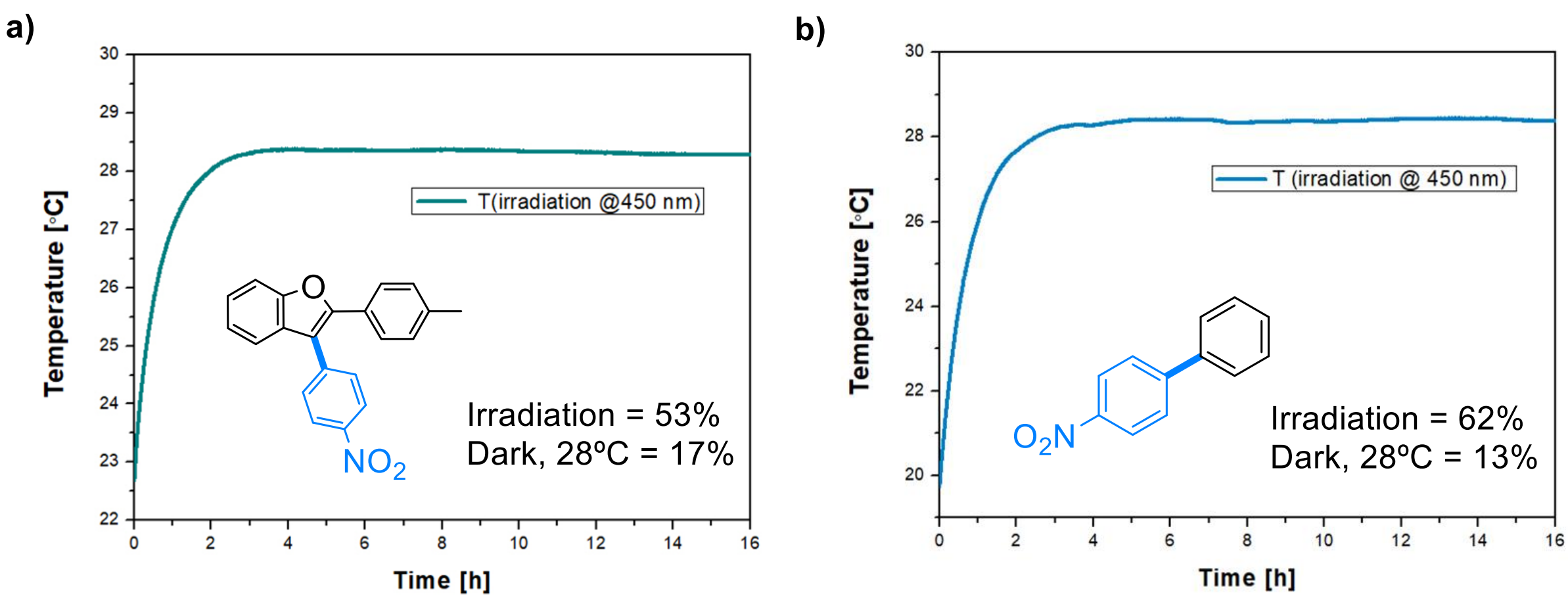

Figure S26. Temperature monitoring for a) arylative cyclization and b) diarylation reactions.

E/Z photoisomerization

We also explored the reactivity of **1** as an EnT-catalyst for the photoisomerization of dimethyl fumarate to dimethyl maleate as reported by Melchiorre and coworkers.[11b]. A mixture of dimethyl fumarate (0.1 mmol, 14.41 mg) and 1 (10 mol%, 0.01 mmol, 7.74 mg) was dissolved in 0.5 mL of freshly degassed MeCN and irradiated at 365 nm for 12 h. The crude was filtered through Celite, evaporated, and redissolved in 0.6 mL of $CDCl_3$. Inspection of the ^{1}H NMR spectrum revealed only the presence of the starting material.

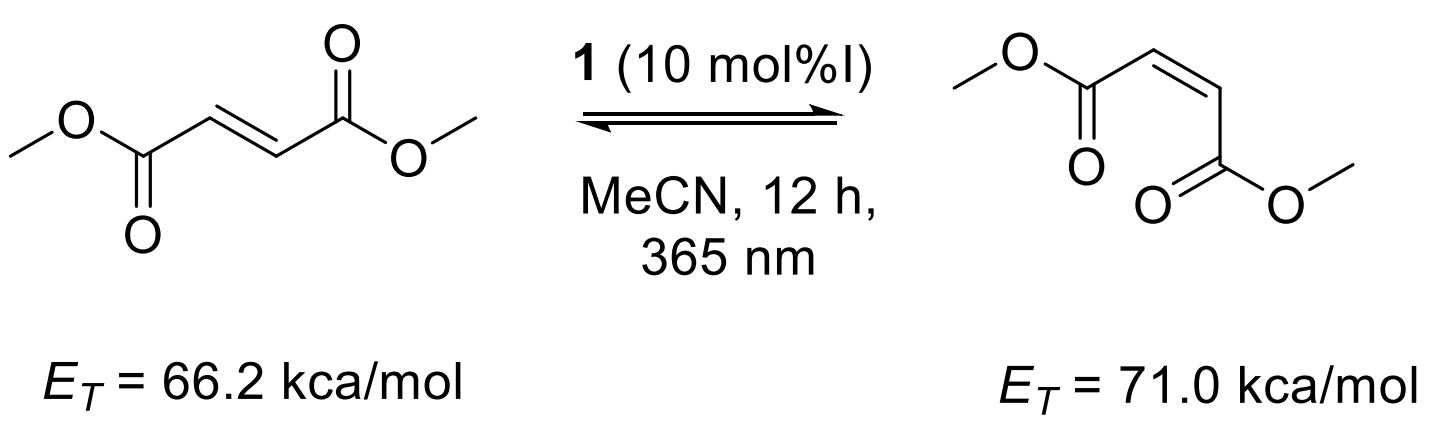

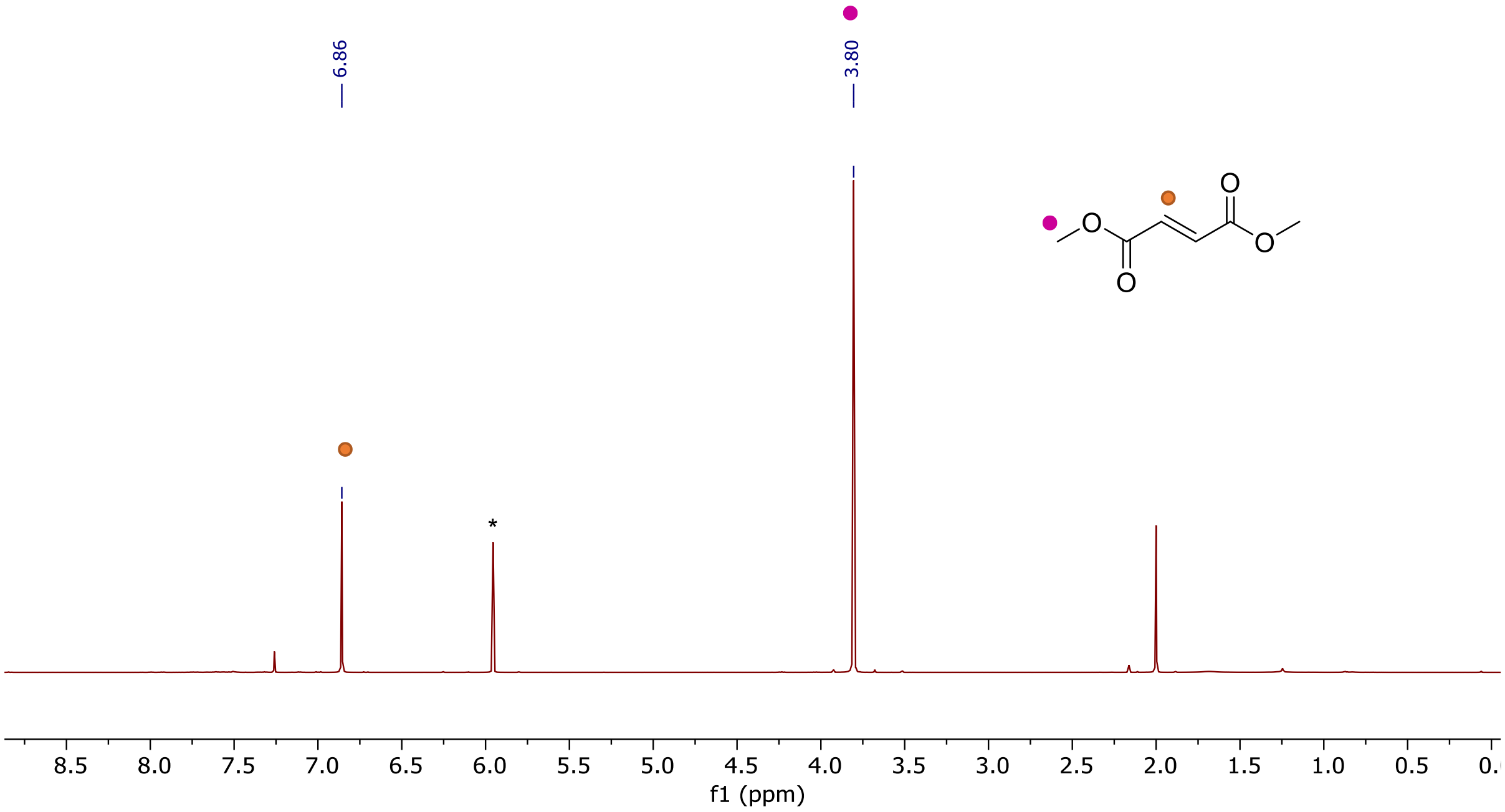

Figure S27. ^{1}H NMR of the reaction between methyl fumarate and **1** (10 mol%) in $CDCl_3$, (*) signal of the internal standard (TTCE).

DFT calculations

Density functional theory (DFT) calculations were carried out in Gaussian 16 Revision C.02[14] and the results were visualized using GaussView 6.06.16[15] or Avogadro.[16] Ground state geometry optimizations were performed in the gas phase at the TPSS[17]-D3(BJ)[18]/def2-TZVP[19] level of theory using the density fitting generated from Gaussian 16 (auto-fitting)[20] in combination with an ultrafine grid. For all calculations, the core electrons of Au (60 electrons) and I (46 electrons) were replaced with effective core potentials (ECPs).[21] Basis sets and ECPs were obtained from the Basis Set Exchange Library.[22] The analytical second derivatives were computed to confirm that minima on the potential energy surface were reached, displaying no imaginary frequencies. Using optimized geometries, TDDFT calculations were performed at the CAM-B3LYP[23]/def2-TZVP[19] level of theory. Solvation effects (MeCN) were simulated with the continuum polarized model (CPM).[24] Natural Transition Orbitals (NTOs)[25] were used to analyze TDDFT calculations.

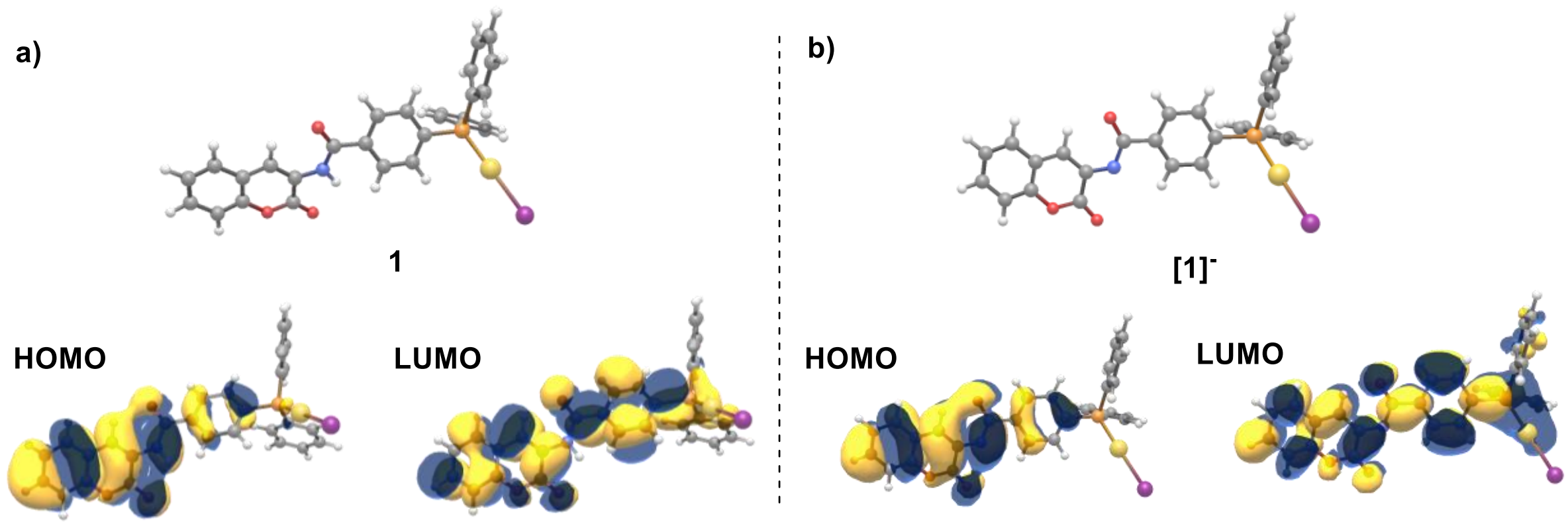

Figure S28. Optimized structures and frontier orbitals (isosurface = 0.03) for a) **1** and b) **[1]**$^{-}$.

Table S5. Comparison between representative bond lengths obtained from X-ray diffraction vs computed values.

Complex	Bond length (Å)	Experimental (Å)	Theoretical (Å)
**1**	Au1-I1	2.5658(5)	2.606
	Au1-P1	2.252(1)	2.267
**[1]**$^{-}$	Au1-I1		2.606
	Au1-P1		2.269

TDDFT

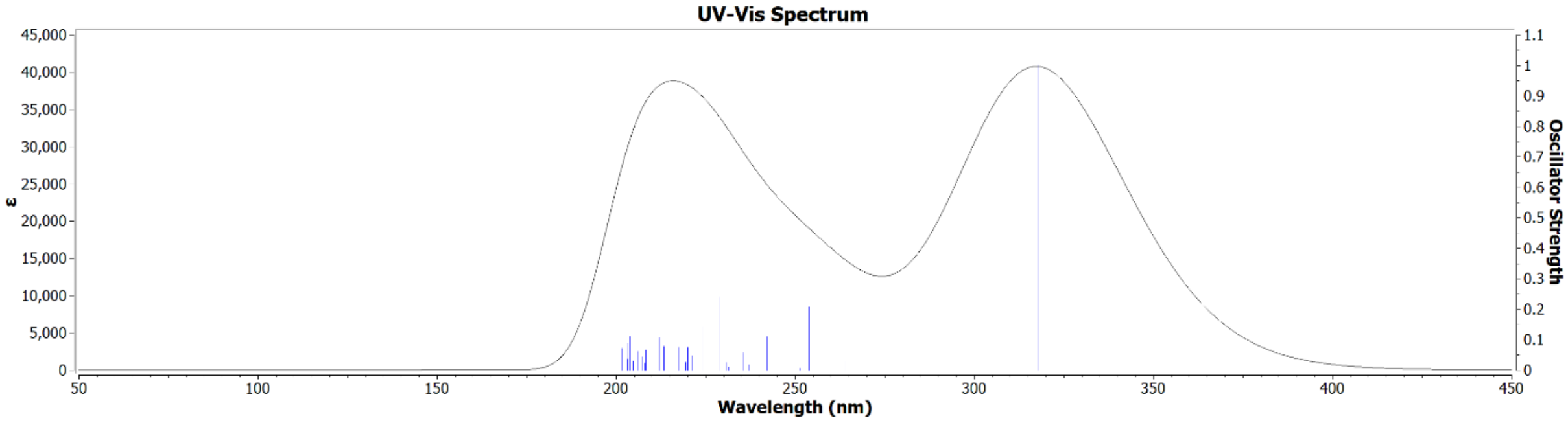

Figure S29. Computed UV-vis spectrum for **1** based on the calculated oscillator strengths of the first thirty vertical singlet excitations (CAM-B3LYP/def2-TZVP/CPM(MeCN)).

Table S6. Vertical singlet excitations for the first five excited states.

<table>
<tr><th>Excited state</th><th>Electronic transitions</th><th>λ_{theo} (nm)/Energy (eV)</th><th>λ_{exp} (nm)</th><th>Oscillator strength</th></tr>
<tr><td>1</td><td>H → L
H → L+1</td><td>317.65/3.9032</td><td>328</td><td>1.0001</td></tr>
<tr><td>2</td><td>H-11 → L
H-4 → L
H-4 → L+1
H-3 → L
H → L + 1
H → L + 7</td><td>268.87/4.6113</td><td rowspan="4">238</td><td>0.0899</td></tr>
<tr><td>3</td><td>H-12 → L
H-11 → L
H-4 → L
H-4 → L + 1
H-3 → L + 1
H → L + 1
H → L+ 8</td><td>263.84/4.6993</td><td>0.0271</td></tr>
<tr><td>4</td><td>H-12 → L
H-11 → L
H-4 → L
H-4 → L+1
H-3 → L
H-3 → L+1
H → L+1
H → L + 8</td><td>253.86/ 4.8839</td><td>0.2073</td></tr>
<tr><td>5</td><td>H-11 → L
H-10 → L
H-10→ L+1
H-9 → L
H-9 → L+1
H-7 → L
H-7 → L+1
H-6 → L
H-6 → L+1
H-3 → L + 3
H-3→ L+4
H → L+1</td><td>251.18/4.9360</td><td>0.0075</td></tr>
</table>

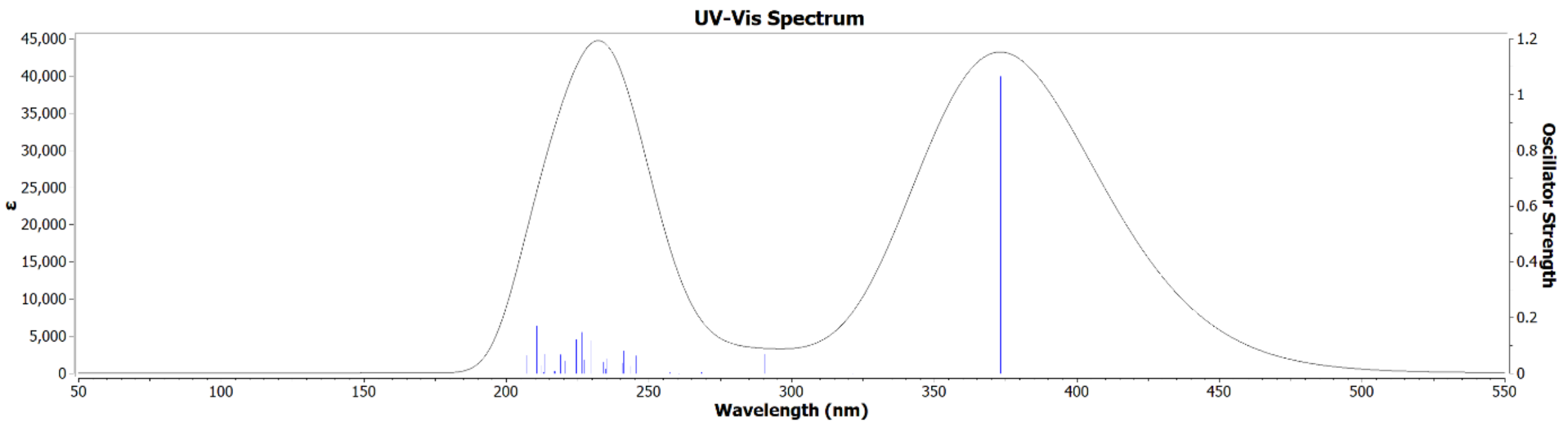

Figure S30. Computed UV-vis spectrum for **[1]**$^-$ based on the calculated oscillator strengths of the first thirty vertical singlet excitations (CAM-B3LYP/def2-TZVP/CPM(MeCN)).

Table S7. Vertical singlet excitations for the first five excited states.

Excited state	Electronic transitions	λ_{theo} (nm)/Energy (eV)	λ_{exp} (nm)	Oscillator strength
1	H → L<br>H → L+3	373.19/3.3223	383	1.0651
2	H-1 → L<br>H-1 → L+16	321.25/3.8594	272	0.0001
3	H-4 → L<br>H → L<br>H → L+2<br>H → L+3<br>H→ L + 4<br>H → L+6<br>H → L+7<br>H → L+11	290.39/4.2696		0.0686
4	H-5 → L<br>H-4 → L<br>H-4 → L + 3<br>H → L+2<br>H → L + 6<br>H → L+7<br>H → L+8<br>H → L + 10	268.33/ 4.6205		0.0032
5	H-8 → L<br>H-7 → L<br>H-1→ L+3<br>H-1 → L + 4	260.49/4.7597		0.0002

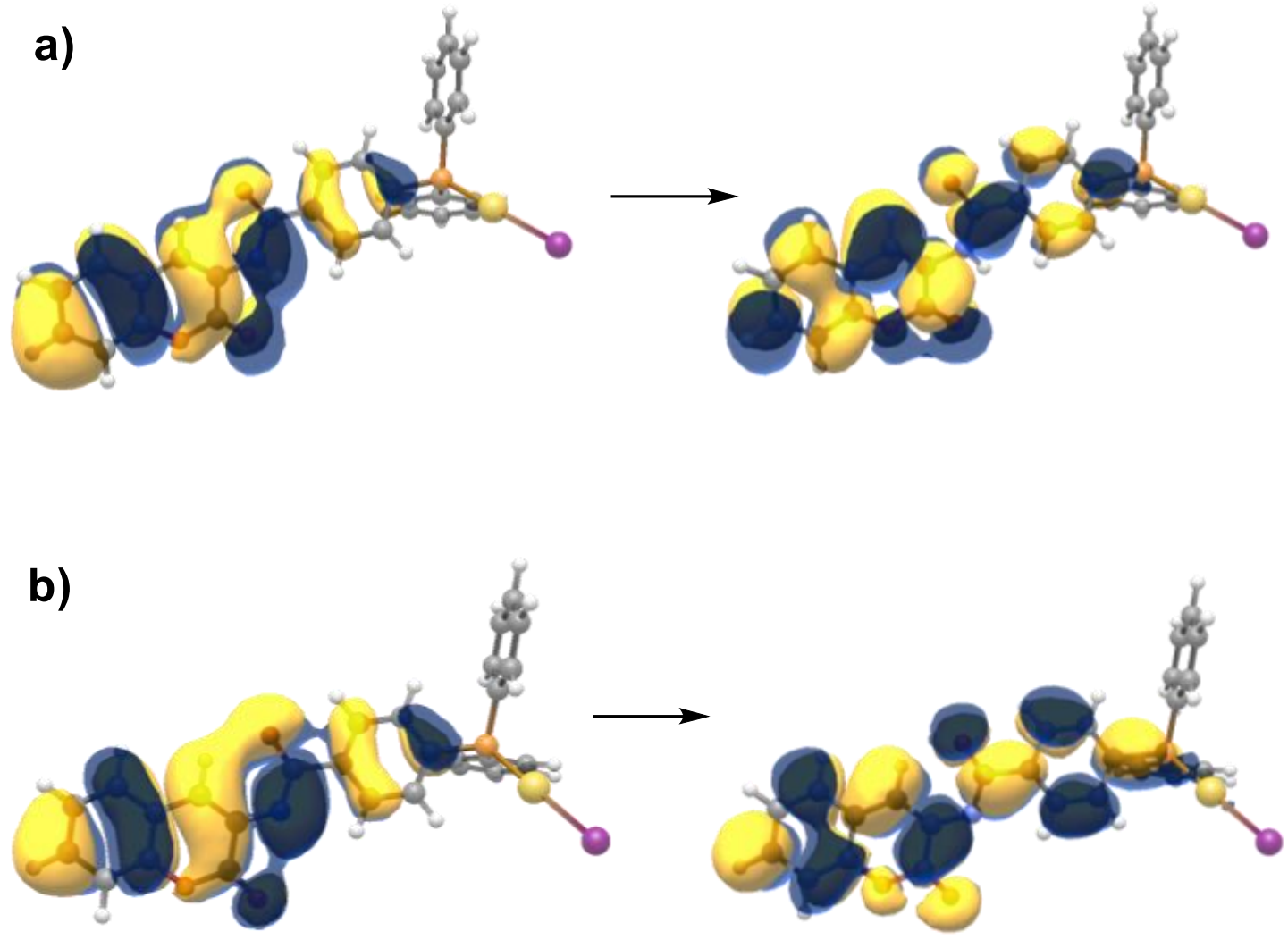

Figure S31. Natural transition orbitals for the first excited state (0.03) in a) **1** and b) **[1]**$^{-}$ represented as a hole → electron.

References

[1] Brandys, M.-C.; Jennings, M. C.; Puddephatt, R. J. Luminescent gold(I) macrocycles with diphosphine and 4,4′-bipyridyl ligands. *J. Chem. Soc. Dalton Trans.* **2000**, (24), 4601-4606.
[2] (a) Kriechbaum, M.; List, M.; Himmelsbach, M.; Redhammer, G. J.; Monkowius, U. Peptide coupling between amino acids and the carboxylic acid of a functionalized chlorido-gold(I)-phosphane. *Inorg. Chem.* **2014**, *53* (19), 10602-10610. (b) Schmidbaur, H.; Brachthhauser, B.; Gamper, S.; Schier, A.; Steigelmann, O. Substituenteneinflusse auf Synthese und Eigenschaften von Hexakis[phosphanaurio(I)methanium(2+)bis(tetrafluoroboraten). *Naturforsch. B.* **1992**, *47*, 1725-1735.
[3] (a) Sharma, R. K.; Priyanka; Katiyar, D. l-Proline catalyzed condensation of salicylaldehydes with ethyl nitroacetate: an efficient access to 3-nitrocoumarins. *Monatsh Chem.* **2016**, *147* (12), 2157-2161. (b) Sharma, R. K.; Singh, V.; Tiwari, N.; Butcher, R. J.; Katiyar, D. Synthesis, antimicrobial and chitinase inhibitory activities of 3-amidocoumarins. *Bioorg. Chem.* **2020**, *98*, 103700.
[4] Taschinski, S.; Dopp, R.; Ackermann, M.; Rominger, F.; de Vries, F.; Menger, M.; Rudolph, M.; Hashmi, A. S. K.; Klein, J. Light-Induced Mechanistic Divergence in Gold(I) Catalysis: Revisiting the Reactivity of Diazonium Salts. *Angew. Chem. Int. Ed.* **2019**, *58* (47), 16988-16993.
[5] Sheldrick, G. Crystal structure refinement with SHELXL. *Acta Cryst.* **2015**, *C71* (1), 3-8.
[6] Mauricio, M.-B.; Michel, L.; Juan Diego, M.; Betty, M.; Claudio, O.-A.; Giovanna, L. D.; Eugenio, U.; Lourdes, S.; Maria Joao, M. Evaluation of Trypanocidal and Antioxidant Activities of a Selected Series of 3-amidocoumarins. *Med. Chem.* **2018**, *14* (6), 573-584.
[7] Thasana, N.; Worayuthakarn, R.; Suddee, N.; Nealmongkol, P.; Ruchirawat, S. Copper-Mediated C–O/C–N Bond Formation: A Facile Synthesis of 3-Amidocoumarin, 3-Amidoazacoumarin, and N-Aroylindole -Derivatives. *Synlett* **2022**, *33* (14), 1431-1437.
[8] *SADABS, Program for Empirical Absorption Correction of Area Detector Data*; University of Göttigen: Germany, 2014.
[9] Sheldrick, G. SHELXT - Integrated space-group and crystal-structure determination. *Acta Cryst.* **2015**, *A71* (1), 3-8.
[10] Spek, A. L. Platon Squeeze. *Acta Cryst.* **2009**, *D65*, 148-155.
[11] (a) Strieth-Kalthoff, F.; James, M. J.; Teders, M.; Pitzer, L.; Glorius, F. Energy transfer catalysis mediated by visible light: principles, applications, directions. *Chem. Soc. Rev.* **2018**, *47* (19), 7190-7202. (b) Mallik, S.; Wang, H.; Matera, N.; Li, B. X.; Stagni, S.; Melchiorre, P. A Bifunctional Organic Photocatalyst for Efficient Single-Electron and Energy Transfer Activation. *Angew. Chem. Int. Ed.* **2025**, e202509770.
[12] (a) Kolb, D.; Morgenstern, M.; Konig, B. Decarbonylation of benzaldehydes by dual photoorgano-cobalt catalysis. *Chem. Commun.* **2023**, *59* (55), 8592-8595. (b) Schroll, P. Photocatalytic Single-Electron Transfer Reactions: The Photo-Meerwein Arylation. PhD Thesis, Universität Regensburg, Germany, 2014.
[13] Cornilleau, T.; Hermange, P.; Fouquet, E. Gold-catalysed cross-coupling between aryldiazonium salts and arylboronic acids: probing the usefulness of photoredox conditions. *Chem. Commun.* **2016**, *52* (65), 10040-10043.
[14] *Gaussian 16 Rev. C.02*; Wallingford, CT, 2016.
[15] *GaussView 6.06.16*; Semichem Inc.: Shawnee Mission, KS, 2016.
[16] Hanwell, M. D.; Curtis, D. E.; Lonie, D. C.; Vandermeersch, T.; Zurek, E.; Hutchinson, G. R. Avogadro: an advanced semantic chemical editor, visualization, and analysis platform. *J. Cheminform.* **2012**, *4*, 1-17.
[17] Tao, J.; Perdew, J. P.; Staroverov, V. N.; Scuseria, G. E. Climbing the density functional ladder: nonempirical meta-generalized gradient approximation designed for molecules and solids. *Phys. Rev. Lett.* **2003**, *91* (14), 146401.
[18] (a) Grimme, S.; Antony, J.; Ehrlich, S.; Krieg, H. A consistent and accurate ab initio parametrization of density functional dispersion correction (DFT-D) for the 94 elements H-Pu. *J. Chem. Phys.* **2010**, *132* (15), 154104. (b) Schroder, H.; Creon, A.; Schwabe, T. Reformulation of the D3(Becke-Johnson) Dispersion Correction without Resorting to Higher than C(6) Dispersion Coefficients. *J. Chem. Theory Comput.* **2015**, *11* (7), 3163-3170.
[19] Weigend, F.; Ahlrichs, R. Balanced basis sets of split valence, triple zeta valence and quadruple zeta valence quality for H to Rn: Design and assessment of accuracy. *Phys. Chem. Chem. Phys.* **2005**, *7* (18), 3297-3305.
[20] (a) Dunlap, B. I. Fitting the Coulomb potential variationally in Xα molecular calculations. *J. Chem. Phys.* **1983**, *78* (6), 3140-3142. (b) Dunlap, B. I. Robust and variational fitting: Removing the four-center integrals from center stage in quantum chemistry. *J. Mol. Struct. THEOCHEM* **2000**, *529*, 37-40.
[21] Titov, A. V.; Mosyagin, N. S. Generalized relativistic effective core potential: Theoretical grounds. *Int. J. Quantum Chem.* **1999**, *71* (5), 359-401.

[22] Pritchard, B. P.; Altarawy, D.; Didier, B.; Gibson, T. D.; Windus, T. L. A New basis Set Exchange: An Open, Up-to-date Resource for the Molecular Sciences Community. *J. Chem. Inf. Model.* **2019**, *59* (11), 4814-4820.
[23] Yanai, T.; Tew, D. P.; Handy, N. C. A new hybrid exchange–correlation functional using the Coulomb-attenuating method (CAM-B3LYP). *Chem. Phys. Lett.* **2004**, *393* (1-3), 51-57.
[24] Barone, V.; Cossi, M. Quantum Calculation of Molecular Energies and Energy Gradients in Solution by a Conductor Solvent Model. *J. Phys. Chem. A* **1998**, *102*, 1995-2001.
[25] Martin, R. L. Natural Transitions Orbitals. *J. Chem. Phys.* **2003**, *118*, 4775–4777.
